# Eukaryotic Protein Kinases (ePKs) of the Helminth Parasite *Schistosoma mansoni*

**DOI:** 10.1186/1471-2164-12-215

**Published:** 2011-05-06

**Authors:** Luiza F Andrade, Laila A Nahum, Lívia GA Avelar, Larissa L Silva, Adhemar Zerlotini, Jerônimo C Ruiz, Guilherme Oliveira

**Affiliations:** 1 Genomics and Computational Biology Group, Instituto Nacional de Ciência e Tecnologia em Doenças Tropicais, Centro de Pesquisas René Rachou, Fundação Oswaldo Cruz - FIOCRUZ, Belo Horizonte, MG- 30190-002, Brazil; 2 Centro de Excelência em Bioinformática, Fundação Oswaldo Cruz - FIOCRUZ, Belo Horizonte, MG- 30190-110, Brazil; 3 Instituto de Ciências Biológicas, Universidade Federal de Minas Gerais - UFMG, Belo Horizonte, MG- 31270-910, Brazil

## Abstract

**Background:**

Schistosomiasis remains an important parasitic disease and a major economic problem in many countries. The *Schistosoma mansoni *genome and predicted proteome sequences were recently published providing the opportunity to identify new drug candidates. Eukaryotic protein kinases (ePKs) play a central role in mediating signal transduction through complex networks and are considered druggable targets from the medical and chemical viewpoints. Our work aimed at analyzing the *S. mansoni *predicted proteome in order to identify and classify all ePKs of this parasite through combined computational approaches. Functional annotation was performed mainly to yield insights into the parasite signaling processes relevant to its complex lifestyle and to select some ePKs as potential drug targets.

**Results:**

We have identified 252 ePKs, which corresponds to 1.9% of the *S. mansoni *predicted proteome, through sequence similarity searches using HMMs (Hidden Markov Models). Amino acid sequences corresponding to the conserved catalytic domain of ePKs were aligned by MAFFT and further used in distance-based phylogenetic analysis as implemented in PHYLIP. Our analysis also included the ePK homologs from six other eukaryotes. The results show that *S. mansoni *has proteins in all ePK groups. Most of them are clearly clustered with known ePKs in other eukaryotes according to the phylogenetic analysis. None of the ePKs are exclusively found in *S. mansoni *or belong to an expanded family in this parasite. Only 16 *S. mansoni *ePKs were experimentally studied, 12 proteins are predicted to be catalytically inactive and approximately 2% of the parasite ePKs remain unclassified. Some proteins were mentioned as good target for drug development since they have a predicted essential function for the parasite.

**Conclusions:**

Our approach has improved the functional annotation of 40% of *S. mansoni *ePKs through combined similarity and phylogenetic-based approaches. As we continue this work, we will highlight the biochemical and physiological adaptations of *S. mansoni *in response to diverse environments during the parasite development, vector interaction, and host infection.

## Background

Human schistosomiasis caused by blood fluke parasites of *Schistosoma *genus, remains an important parasitic disease and a major health economic problem in many tropical and subtropical countries. Schistosomes have a complex life cycle that includes six different stages (cercariae, schistosomula, adult worms - male and female, egg, miracidia and sporocyst) in different environments: water, definitive host (mammals) and intermediate host (snail). During parasite development, signals from the environment are sensed and stimulate physiological, morphological and, biochemical adaptations. Oils are shown to stimulate cercarial penetration; hormones and exposure to the snail haemolymph trigger specific physiological adaptations [1-3]. The free living parasite forms display light and geotropism and female development is dependent on signals from the male adult worm through mechanisms not completely understood [4,5]. It has been demonstrated that worm pairing induces changes in gene expression in the female vitelline gland [4] and the accumulation of glutathione and lipids in the male [5]. Furthermore, microarray analysis revealed distinct differential gene expression profiles between males and females [6-8]. Therefore, the success of the parasite infection depends on the assessment at the cellular and molecular levels of the environment and the transmission of signals to physiological regulatory networks that will collectively stimulate adaptations.

The maintenance of homeostasis and complex cellular adaptations in *Schistosoma mansoni *require specific extracellular signals that must be integrated to generate an appropriate response from the sensory receptor via intracellular proteins [3]. Signal transduction involves non-linearly integrated networks that interact mostly by switching activity status via phosphorylation (protein kinases) and dephosphorylation (protein phosphatases) of amino acid residues, or the incorporation of GTP. Other cellular non-protein messengers include cyclic AMP, Ca ^2+ ^and diacylglycerol.

Protein kinases (PKs) play a central role in mediating intracellular signals by adding a phosphate group from ATP or GTP to an amino acid residue leading to a conformational change in the target protein that will switch its activation status [9]. Most PKs have a catalytic domain, which binds and phosphorylates target proteins, and a regulatory region. Many PKs are autophosphorylated or may be phosphorylated by other PKs, an interaction regulated by the accessory protein domains [10].

PKs are classified into two superfamilies containing the eukaryotic or conventional protein kinases (ePK) that share a conserved catalytic domain, and the atypical protein kinases (aPKs) (Table 1). The catalytic domain of ePKs is composed of 250-300 amino acids and is divided into 12 subdomains with highly conserved individual amino acids and motifs [11]. aPKs are reported to have biochemical kinase activity, but lack sequence similarity to the ePK catalytic domain [12]. According to their substrate recognition sites, ePKs are divided broadly into two major classes, serine/threonine kinases (STKs) and tyrosine kinases (TKs). Dual specificity kinases (Hybrid), which phosphorylate serine, threonine, and tyrosine, are also found. ePKs have been further classified into eight groups based on sequence similarity of their catalytic domains, the presence of accessory domains, and their modes of regulation [9,12-14]. According to KinBase [15], a database that holds information of PKs encoded in the human genome and their homologs in other eukaryotes, the eight ePK groups are: AGC (cAMP-dependent protein kinase/protein kinase G/protein kinase C extended), CAMK (Calcium/Calmodulin regulated kinases), CK1 (Cell Kinase I), CMGC (Cyclin-dependent Kinases and other close relatives), RGC (Receptor Guanylate Cyclases), STE (MAP Kinase cascade kinases), TK (Protein Tyrosine Kinase) and TKL (Tyrosine Kinase Like). A ninth group, called ''Other'', consists of a mixed collection of kinases that cannot be classified easily into the previous families [14] (Table 1).

**Table 1:** Protein kinase classification

Superfamily	Class	Group
	Serine/Threonine Kinases	AGC
		CaMK
		CMGC
		STE
		CK1
**Eukaryotic Protein Kinases (ePKs)**		
	Tyrosine Kinases	TK
		RGC
	
	Hybrid	TK like
		Other

Protein kinases are broadly classified into two major superfamilies (ePKs and aPKs). The ePKs groups, families, and subfamilies adopted in the present work followed the proposed hierarchy described elsewhere [15,16,27].

PKs are considered druggable targets from the medical and chemical viewpoints as a growing number of PKs inhibitors have been developed and approved for treatment of different human disease [16]. An example of a successful PK inhibitor is Gleevac^®^, that induces a conformational change in PTK and mimics substrate binding and therefore prevents activation by upstream kinases [17]. Beyond this, PKs have gained interest as targets treatment strategies to fight many parasites, including *S. mansoni *[18-21].

The current schistosomiasis treatment frequently does not cure 100% of those treated in high-risk communities and the emergence of *Schistosoma *resistant strains is a real possibility [22-25]. Thus, the identification of potential drug targets should be further emphasized. The recent sequencing of *S. mansoni *genome and large-scale transcriptome projects have yielded crucial information to the identification of new candidate drugs [26-29]. Understanding protein structure and function in many model organisms can help elucidate the function of their parasite homologs and further enable the application of such information in drug design and development. The study of the kinase complement (kinome) is therefore of major importance for the understanding of the physiology of the organism and also provides insights into how to disrupt the fine adaptative mechanisms. The present work aimed at analyzing the *S. mansoni *predicted proteome data in order to identify all ePKs encoded in the genome of this parasite. For this purpose, we combined computational approaches such as sequence similarity searches using Hidden Markov Models (HMMs) and distance-based phylogenetic analyses. The functional annotation was performed mainly to yield insights into the signaling process related to the complex lifestyle of *S. mansoni*.

## Results and discussion

### The *Schistosoma mansoni *ePKinome

**Figure 1:**
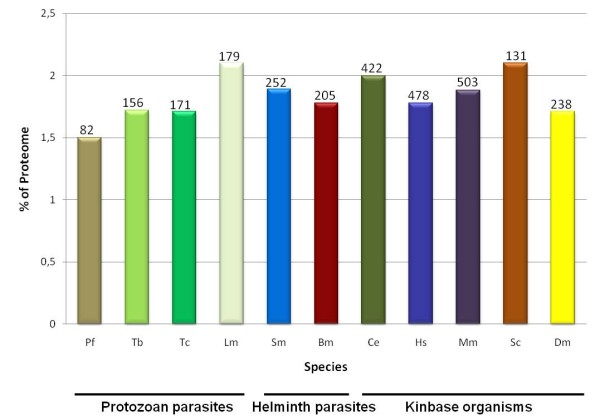
**ePKinome in the predicted proteomes of diverse taxa**. A total of 252 PKs were identified in the predicted proteome of *S. mansoni*. For comparison, the percentage (%) of the total predicted proteome that codes for kinases and the total number of ePKs (shown on top of each bar) is shown for four protozoan parasites: Pf - *Plasmodium falciparum *[107]; Tc - *Trypanosoma cruzi*, Tb - *Trypanosoma brucei*, Lm *- Leishmania major *[89]; two helminth parasites: Bm - *Brugia malayi *[108] and *S. mansoni *[26]; and five model organisms of KinBase Ce - *Caernorhabditis elegans *[51], Hm - *Homo sapiens *[13], Mm - *Mus musculus *[109], Dm - *Drosophila melanogaster *[110] and Sc - *Saccharomyces cerevisiae *[10].

The ePK complement of *S. mansoni*, defined as the ePKinome, was identified by searching the parasite predict proteome with a HMM profile of the ePK catalytic domain of five selected organisms. This analysis revealed 252 ePKs in the *S. mansoni *predicted proteome, representing 1.9% of the total proteins encoded in the parasite genome. Although the total number of protein kinases found across the analyzed species varies greatly (from 82 to 503), the percentage values in respect to the genomes of protozoan and helminth parasites as well as other eukaryotes from KinBase range only between 1.5 to 2% (Figure 1).

Amino acid sequences corresponding to the conserved catalytic domain of ePKs were aligned by MAFFT [30] and further used in phylogenetic analysis based on a distance method as implemented in PHYLIP [31]. The dataset for each ePK group also included the ePK homologs from six other eukaryotes: *Homo sapiens*, *Mus musculus*, *Drosophila melanogaster*, *Caenorhabditis elegans*, *Saccharomyces cerevisiae*, and *Brugia malayi*. This approach allowed us to classify the *S. mansoni *ePKinome at the group, family, and/or subfamily levels based on the hierarchy proposed elsewhere [12,13,32,32], and sometimes provided insights into kinase function and evolution. Detailed information is available in the Additional file 1 that contains, among other things, all *S. mansoni *ePKs with the corresponding identifier from the genome project linked to SchistoDB database [29]. SchistoDB http://www.schistodb.net allows the community to access to all sequences, annotations and other data types integrated into the genomic information. It also provides several tools to analyze retrieve and display the data. In the SchistoDB it is possible to encounter, for each ePK, the development expression stages by EST evidence, information about orthologs, Gene Onthology (GO) function, metabolic pathways, structural information, PDB structures, and links to external databases such as the TDR database [33]. The TDR database contains additional information for *S. mansoni *genes like antigenicity, essentiality, phenotypes and associated compounds (druggability).

**Figure 2:**
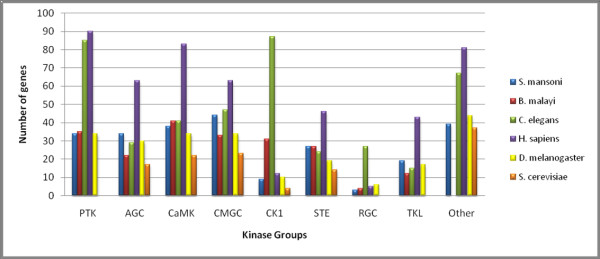
**Distribution of ePKs groups in *S. mansoni *and model organisms**. *S. mansoni *proteins were classified according to KinBase [15] by combining sequence searches (HMMs) and phylogenetic analysis. For comparison, occurrence of the ePKs in *B. malayi, C. elegans*, *H. sapiens*, *D. melanogaster*, *S. cerevisiae*, and is shown. The ePK groups include: PTK (Protein Tyrosine Kinase), AGC (cAMP-dependent protein kinase/protein kinase G/protein kinase C extended), CaMK (Calcium/Calmodulin regulated kinases), CMGC (Cyclin-dependent Kinases and other close relatives), CK1 (Cell Kinase I), STE (MAP Kinase cascade kinases), RGC (Receptor Guanylate Cyclases), TKL (Tyrosine Kinase Like), and Other.

As shown in Figure 2, *S. mansoni *proteins have representatives in the main ePK groups. ePKs that do not fall into these groups are categorized as "Other" in which multiple families have been defined. The *S. mansoni *largest ePK group is CMGC, a feature unique to this parasite, and the smallest group is RGC, a common feature shared with many of the analyzed organisms (Figure 2).

Of the 252 ePKs identified in *S. mansoni *proteome, only 16 were experimentally studied as highlighted in the supplementary material (Additional file 1) and the others 236 ePKs were previously annotated only by automatic methods based on sequence similarity searches [26,29].

*S. mansoni *ePKs were examined for the presence of the 12 smaller subdomains present in the catalytic domain and also for the presence of a lysine in subdomain II and aspartic acids residues in subdomain VIb and VII, which are known to play essential roles in the kinase function [9,12,34]. According to our analysis, 12 proteins are predicted to be catalytically inactive ePKs, as they lack one or more of the three essential amino acid residues in the catalytic domain (Additional file 1), including all members of *S. mansoni *RGC group (see below).

Approximately 2% of the *S. mansoni *ePK remain unclassified once they do not have similarity to any known PK family. All these proteins have a truncated catalytic domain probably because of an incorrect protein prediction. The unclassified ePKs from *C. elegans, D. melanogaster, H. sapiens *and *S. cerevisiae *range from 19% to about 38% their kinomes.

### Serine/Threonine kinases

#### AGC group

Around 13 families have been classified as part of the AGC group in eukaryotic organisms [11]. In *S. mansoni*, most AGC proteins belong to PKA (Protein Kinase A) (5 proteins), DMPK (Myotonic Dystrophy Protein Kinase) (4 proteins and 1 product of alternative splicing), PKC (Protein Kinase C) (4 proteins) and PKG (4 proteins) families. Other *S. mansoni *proteins have only one representative in the remaining AGC families (Additional file 1). According to our phylogenetic analysis, *S. mansoni *has no homolog of the YANK (Yet Another Novel Kinase) family (Additional file 2).

The most significant difference between PKA and PKG family members is that in PKA, the regulatory and catalytic activities are performed by separate gene products known as PKA-R and PKA-C, respectively, whereas in PKG the cNMP-binding (cyclic nucleotide-binding domain) and catalytic domains are usually present in the same polypeptide [35]. The inactive conformation of PKA is a heterotetramer of two PKA-R and two PKA-C subunits, while PKG exists as a homodimer [35]. *S. mansoni *processes five homologs of the PKA-C subunit (Additional file 1), and six predicted of PKC-R subunit (Smp_131050, Smp_147320, Smp_079010, Smp_030400, Smp_019280, Smp_022100) allowing for a variety of different holoenzymes to be formed in this parasite. Some studies demonstrated that PKG proteins of *Toxoplasma *[36] and *Eimeria *[37] and PKG and PKA proteins of *Plasmodium *[38,39] are essential as the inhibitors causes an anti-parasite effect in these organisms. Recently it was shown that inhibition of the SmPKA-C subunit (Smp_152330), expressed in adult worms of *S. mansoni*, resulted in the death of the parasites [40]. This result and the range of holoenzymes that can be formed, indicate that genes in this family are critical for the development of *S. mansoni *and may represent good targets for drug development.

PKC belongs to a large protein family that is classified into four important subfamilies: PKC Alpha subfamily, that contain the conventional PKCs (γ, βI, βII, and α) and are sensitive to diacylglycerol (DAG) and Ca^2+^; PKC Eta and Delta subfamilies containing the *novel *PKCs (ε, δ, η, and θ) which are regulated by DAG alone; and PKC Iota subfamily, that contain the atypical PKCs (ζ and ι), and are insensitive to both compounds [41,42]. PKC is considered to be a mechanistic regulator of development in vertebrates, playing a key role in cell growth and differentiation [43-45]. *S. mansoni *has representatives in the three main PKC subfamilies mentioned above (Iota, Eta and Alpha) but lacks homologs in the Delta subfamily, present in *C. elegans*, *D*. *melanogaster, M. musculus*, and *H. sapiens*. The two PKC Alpha proteins found in *S. mansoni *(Smp_128480 and Smp_176360), belong to the PKCβI isoform and were recently characterized [46-48]. Both are associated with the neural mass, excretory vesicle, ridge cyton, tegument and germinal cells in schistosomula and miracidium, suggesting a possible role in larval transformation [46-48].

One protein in AGC group, Smp_157370, remains unclassified. In the phylogenetic tree, this protein appears more closely related to the GRK (G-protein coupled Receptor Kinase) family (Additional file 2), despite the good conservation of the catalytic domain, this protein lacks the accessory domain that is characteristic of the GRK proteins (Additiona file 2). Furthermore, Smp_157370 does not form a clade with the GRK family members according to our phylogenetic tree, which corroborates its divergence in relation to GRK homologs in other eukaryotes (Additional file 2).

Interestingly, according to SchistoDB [29] EST evidences, the two most highly transcribed ePKs (Smp_151140 and Smp_158560.1) in *S. mansoni*, belong to the DMPK family of the AGC group, mainly in cercariae, schistosomula, eggs and adult worms. This finding is interesting as these are the four life cycle stages of the parasite which are in contact with the definitive host. In *C. elegans p*roteins of DMPK family are expressed in hypodermal cells and are involved in embryonic elongation [49].

#### CaMK group

The divalent cation calcium (Ca^2+^) is one of the ions most widely used as a second messenger in cellular signaling. A significant portion of calcium-mediated signaling is controlled by calmodulin-binding kinases. Some members of the CaMK group are dependent on the binding of Ca^2+^/CaM [50]. In the *S. mansoni *ePKinome, 32 proteins were classified as CaMK with the vast majority (18 proteins) belonging to the CaMKL (Calcium/Calmodulin Regulated Kinase) - like family. A similar number was found in other organisms analyzed here (Additional file 3). *S. mansoni *also contain members of DAPK (death associated protein kinase), MAPKAPK (MAPK associated protein kinase), MLCK (myosin light chain kinase), and PHK (phosphorylase kinase) families in the CaMK group (Additional file 4).

MLCK is a Ca^2+^/calmodulin-dependent protein kinase whose only known substrate is myosin II regulatory light chain [51]. The primary function of MLCK is to stimulate muscle contraction through the phosphorylation of the myosin II regulatory light chain (RLC), a eukaryotic motor protein that interacts with filamentous actin. Although MLCK has only one known substrate (RLC), this protein is linked to a variety of cellular processes due to the diverse biological function of myosin II [50]. Two distinct smooth muscle MLCK genes were identified in *S. mansoni *(Smp_121780, Smp_126240), although no homologs were identified for the non-smooth muscle vertebrate MLCK through our phylogenetic analysis. This likely reflects the absence of a striated muscle in this parasite.

DCAMKL (Doublecortin and CaMK-like) is a protein that regulates the microtubule cytoskeleton and in the chick is specifically expressed in the developing brain [52,53]. CASK is a protein that participates in cell adhesion [54]. According to our phylogenetic analysis, a single homolog of the DCAMKL (Smp_053560) and CASK (Smp_131690) families were found in *S. mansoni *(Additional file 4).

While the CaMK2 (CaMK family 2) family is encoded by four genes in humans, only a single CaMK2 gene, with two predicted alternative spliced transcripts, was identified in the *S. mansoni *genome (Additional file 1). *S. mansoni *CaMK2 was recently identified as putative target for drug development after comparative chemogenomics approach using the *S. mansoni *proteome and the proteome of two model organisms, *C. elegans *and *D. melanogaster*. [55]. The function of this protein in *S. mansoni *is still unknown. In sea urchin, CaMK2 is required for nuclear envelope breakdown following fertilization [56].

#### CMGC group

CMGC kinases are relatively abundant in *S. mansoni*, a feature that can be explained by the requirement to control cell proliferation and to ensure correct replication and segregation of organelles, which together are essential mechanisms for parasites with a complex life cycle. In the CMGC group, all of the main families are conserved between *S. cerevisiae*, *C. elegans*, *M. musculus*, *H. sapiens*, and *S. mansoni*, including CDK (Cyclin Dependent Kinase), MAPK (Mitogen Activated Protein Kinase), GSK (Gycogen Synthase 3 Kinase), CLK (CDC-Like Kinase), SRPK (SR Protein Kinase), CK2 (Cell Kinase 2), and DYRK (Dual-specificity Tyrosine Regulated Kinase) (Additional file 5) and RCK.

*S. mansoni *has 14 CDKs, the same number was found in *C. elegans *(compared with only seven in *S. cerevisiae*), including homologs of all subfamilies (CDK7, CDK4, CDK8, CRK7, CDK9, PITSLRE, CDK10, PCTAIRE, PFTAIRE, VDK5 and CDC2) (Additional file 5). On the other hand, only one RCK family protein (Smp_132890) was identified in the parasite. The RCK proteins are similar to mammalian MAK (male germ cell-associated kinase), which have been implicated in spermatogenic meiosis and in signal transduction pathways for sight and smell [51].

GSK family is represented by 3 proteins in *S. mansoni*. One of those (Smp_008260.1) was selected as putative target for drug development after comparative chemogenomics approach [55]. GSK proteins are involved in development and cell proliferation, are overexpressed in colon carcinomas and positively regulates the Wnt signaling pathway during embryonic development and oocyte-to-embryo transition in *C. elegans *[57].

**Figure 3:**
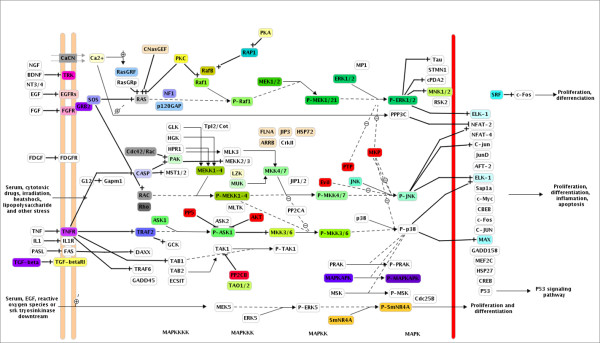
**MAPK signaling pathway**. MAPKs are expressed in all eukaryotic cells and are activated by diverse *stimuli *ranging from cytokines, growth factors, neurotransmitters, hormones, cellular stress, and cell adherence. The basic assembly of the MAPK pathway is a three-component module that includes three kinases that establish a sequential activation pathway comprising a MAPK kinase kinase (MKKK, MEKK, STE11, or STE13), MAPK kinase (MKK, MEK or STE7), and MAPK (green). The activated MAPK may translocate to the nucleus and bind to transcription factors (light blue). The mammalian MAPK can be subdivided into five families: ERK, p38, JNK, nml, and ERK5. Each MAPK family has distinct biological functions. Colored blocks correspond to proteins indentified in the *S. mansoni *predicted proteome and white blocks are mammals' proteins with no homologs in *S. mansoni *predicted proteome. The + signal represents protein activation and - signal protein inhibition by protein phosphatases (red colored proteins). *S. mansoni *has all representatives of ERK, p38, and JNK pathways including proteins of STE (MEK1/2, MKK), AGC (PKC and PKA), CaMK (MAPKAPK), TK (EGFR, FGFR), and TKL (Raf and TGFbeta-RI) groups.

The MAPK signaling pathways are some of the best characterized signaling systems. *S. mansoni *contains nine MAPKs, compared to seven in *D. melanogaster *and 14 in *C. elegans*. As shown in Figure 3, mammals have, at least five MAPK cascades described; these include the extracellular signal-regulated kinase (ERK) cascade, which regulates cell growth and differentiation, the c-Jun N-terminal kinase (JNK)/stress-activated protein kinase (SAPK), and the p38 MAPK cascades, which function mainly in stress responses such as inflammation and apoptosis [58]. In *D. melanogaster *and *C. elegans*, the MAPK pathways are involved in critical cellular and developmental processes [59,60]. *S. cerevisiae *has four distinct MAPK signaling pathways that are likely mediators of responses to pheromone, nutritional starvation, and cellular or osmotic stress [51]. The MAPK signaling pathways are well conserved in *S. mansoni *(Figure 3), including representatives of the subfamilies ERK, p38, JNK, and, NLK but lacks members of ERK5 that are part of a signaling pathways found mainly in mammals (Additional file 5). Each subfamily is activated by different stimuli that generate different biological responses [61-63]. In *S. mansoni *only one protein was identified in JNK (Smp_172240) subfamily. JNK proteins play key roles in human cell function [62] and in the development of *C. elegans *worms [64]. JNK may have an important role in schistosome survival and represent a good target for experimental approaches.

#### STE group

In *S. mansoni*, the STE group includes seven STE7 (MEK or MAPKK), two STE11 (MEKK or MAPKKK), and 13 STE20 (MEKKK) kinases (Additional file 6). The large number of STE family members in *S. mansoni *could translate into an enormous potential for downstream signal specificity and diversity. SmSLK (Smp_150260) is a Ste20 family protein, recently characterized in *S. mansoni*, which is able to activate protein MAPK/JNK in human embryonic kidney (HEK) cells as well as in *Xenopus *oocytes. In addition, imunofluorescence showed that SmSLK was abundant in the tegument of adult schistosomes [65]. These findings indicate that signals sensed in the environment by many different proteins may activate the MAPK cascade that will generate an adaptive physiological response. Futhermore, molecules that activate the MAPK pathways, as some hormone and cytokine signals, are not found in the *S. mansoni *predicted proteome (Figure 3). It has been demonstrated that the parasite takes advantage of host proteins for its growth and development [66,67]. Other ePKs such as members of the PKA, PKC, Raf and receptor protein tyrosine kinases (RTKs) families, also participate in MAPK signaling pathway. RTKs are anchored to the membrane and have an important role in transmitting the signal from the extracellular to cytoplasm (Figure 3) [64].

In *C. elegans *genome studies such as classical forward genetic and RNA interference screens and systematic targeted gene knockout revealed genes that are essential to the organism [68]. Although the off-target and non-specific effect of RNAi [69,70], in *S. mansoni *this is one of the best approaches to explore the functional property of the genes since the knockout experiments are not yet available for schistosomes [71]. By analyzing the phylogenetic trees of the present work, it was possible to identify the proteins of *S. mansoni *that have homologs in *C. elegans *and display lethality and sterile phenotypes by RNAi (Table 2). Interestingly, most essential proteins in table 2 belong to the CMGC and STE groups, suggesting the involvement of these proteins in signaling pathways that culminate in essential cellular processes.

**Table 2:** Orthology relationships among ePKs of *S. mansoni*, *B. malayi*, and *C. elegans *and RNAi phenotype for *C. elegans *proteins

Group	*S. mansoni*	*C. elegans*	*Brugi malayi*	*C. elegans RNAi phenotype*
**CaMK**				
	Smp_053560	C44C8.6_Ce e K08F8.1	**-**	Embryonic Lethal
**AGC**				
	Smp_123640	F19C6.1_Ce	**-**	Embryonic lethal, maternal sterile, organism morphology variant.
	Smp_136750	Y47D3A.16_Ce	Bm1_44635	Larval lethal, fertility reduced
	Smp_158560	K08B12.5_Ce	Bm1_02320	Body morphology defect, slow growth
	Smp_096310	pkc-3_Ce_A	Bm1_03335	Embryonic lethal, sterile, body morphology defect
**TK**				
	Smp_139480	vab-1_Ce_T	Bm1_03410	Embryonic lethal, body morphology defect
**STE**				
	Smp_026510, Smp_151670	mek-2_Ce	**-**	Embryonic lethal, sterile, reduced brood size, exploted through vulva, slow growth
	Smp_096640	gck_1_Ce	**-**	cortical dynamics defective early embryonic, maternal sterile
	Smp_163420	mig_15_Ce	Bm1_32540	Abnormal cell migration, protruding vulva, developmental delay
	Smp_068060	kin_18_Ce	Bm1_55590	Embryonic lethal, body morphology defect, slow growth, sterile, larval lethal, dumpy
	Smp_131800	Y59A8B.23_CE	Bm1_11845	More depolarized oocytes
	Smp_150260	C04A11.3_Ce	Bm1_26435	Slow growth, larval lethal late L3/L4
**TKL**				
	Smp_079760	pat4_Ce_TK	Bm1_20815	Embryonic lethal, body morphology defect, larval lethal, sterile, paralyzed, uncoordinated movement, paralyzed
	Smp_176990	lin45_Ce_T	Bm1_40290	Sterile, osmotic integrity problems
**CMGC**				
	Smp_068960	F22D6.5_Ce	Bm1_12230	Embryonic lethal, slow growth, sterile progeny, uncoordinateed movement
	Smp_041770	spk_1_Ce	**-**	Embryonic lethal, larval lethal, maternal sterile
	Smp_172700	cdk_4_Ce	**-**	Locomotion variant, larval lethal, sterile progeny
	Smp_133020	pmk-1_Ce, pmk-2_Ce	**-**	Apoptosis increased, sterile progeny, reduced brood size, embryonic lethal
	Smp_134260, Smp_133490, Smp_133500	C05D10.2	**-**	Reduced brood size, embryonic lethal
	Smp_080730	cdk_1_Ce	**-**	embryonic lethal, sterile progeny
	Smp_155720, Smp_125310, Smp_008260.1	Y18D10A.5_Ce, C44H4.6_Ce	**-**	Larval lethal, slow growth, embryonic lethal
	Smp_156990	F39H11.3_Ce	Bm1_33825	Sterile
	Smp_176620	B0495.2_Ce, ZC504.3_Ce	Bm1_17240	Embryonic lethal
	Smp_003000	H25P06.2_Ce	**-**	Embryonic lethal, slow growth, uncoordinated movement
	Smp_150040	Cdk_7_Ce	Bm1_14135	Embryonic lethal
	Smp_140700, Smp_074080	lit_1_Ce	Bm1_130	Embryonic lethal, larval lethal
	Smp_141230	mbk_2_Ce	Bm1_38345	Embryonic lethal, sterile progeny

Orthology relationships were inferred based on phylogenetic trees. RNAi phenotype for *C. elegans *were visualized by recent work [68] that relied on identification and analysis of essential genes.

#### CK1 Group

**Figure 4:**
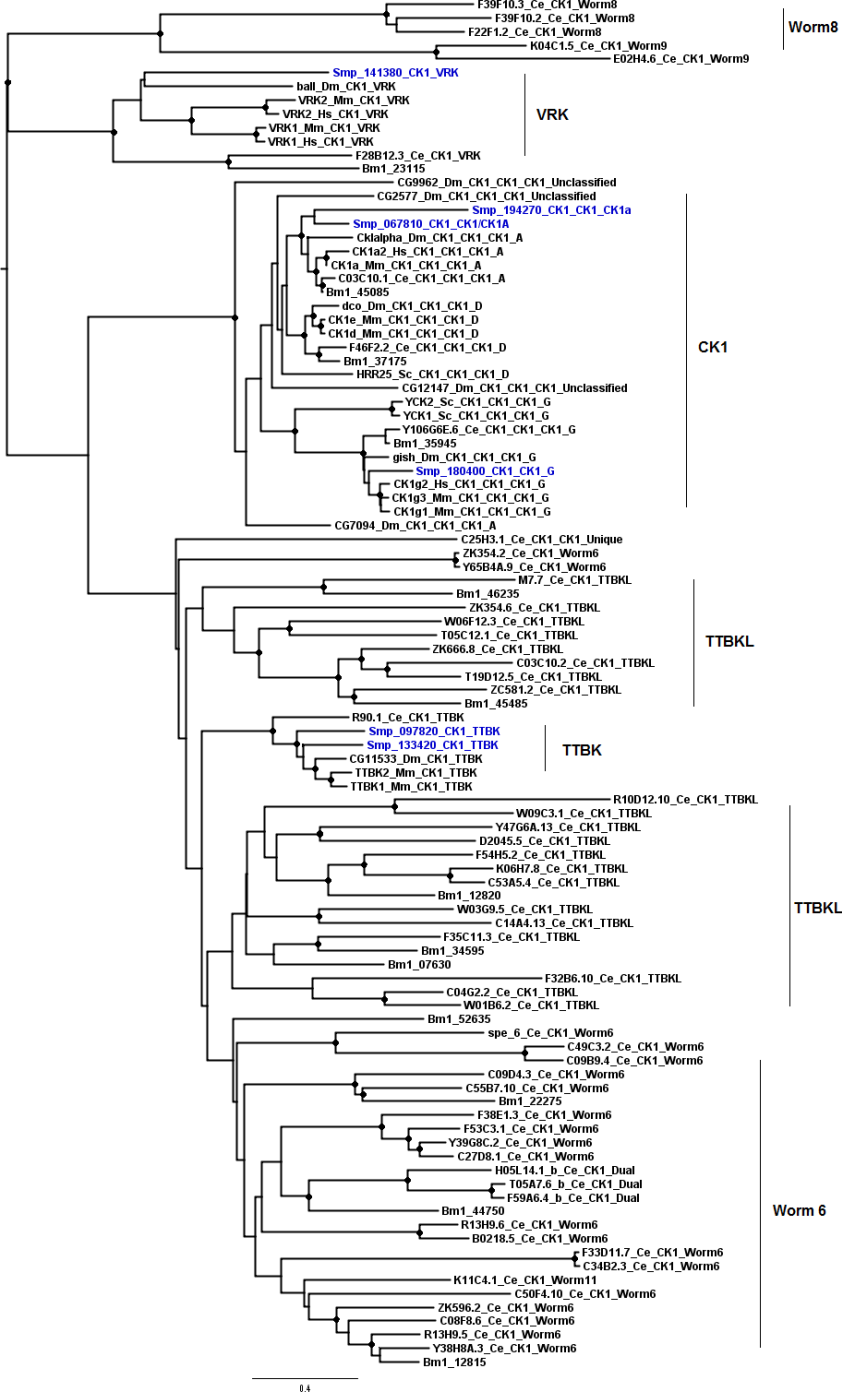
**Phylogenetic analysis of the CK1 group**. Amino acid sequences of the catalytic domain of the CK1 proteins of *S. mansoni *(blue labels), *C. elegans*, *D. melanogaster*, *S. cerevisiae*, *H. sapiens*, and *B. malayi *were used to construct a distance-based phylogenetic tree using PHYLIP programs. Bootstrap values (1000 replicates) equal or higher than 80% are indicated (●) as cutoff values to support the family/subfamily classification. Functional classification is indicated individually in the protein labels. Protein families are highlighted by the vertical bars and include: VRK (Vaccinia Related Kinase), CK1 (Casein Kinase 1), TTBK (Tau Tubulin Kinase), Worm 8 (a specific family of *Caenorhabditis*), TTBKL (TTBK-Like kinase), and worm 6, two specific families of *C. elegans *and *B. malayi*.

The two smallest groups found in the *S. mansoni *ePKinome were CK1 and RGC (Figure 2). In contrast, in *C. elegans *CK1 is the largest group and RGC is dramatically expanded. However, these expansions are a unique feature of *C. elegans*, as compared to other eukaryotes selected for this analysis (Figure 4). The CK1 group consists of three main ePK families: CK1, VRK (Vaccinia Related Kinase), and TTBK (Tau Tubulin Kinase) that formed three individual clusters in the phylogenic tree (Figure 4). *S. mansoni *has representatives in each of these families also found in *C. elegans*, *D. melanogaster*, *M. musculus*, *H. sapiens*, *S. cerevisiae *and *B. malayi *kinomes. The nematodes, *C. elegans *and *B. malayi*, still have two other families that seem to be specific to this taxonomic group, TTBKL and Worm6. The Worm8 family was identified only in *Caenorhabditis *so far. The diversification of the CK1 group in *C. elegans *may be an adaptation allowing for enhanced DNA repair in response to excessive exposure to environmental mutagens [51]. One CK1 encoding gene (*spe-6*) functions in spermatogenesis, and at least half of the proteins in this group are selectively expressed in *C. elegans *sperm as shown by microarray analysis [72,73]. The role of these proteins in the parasite *S. mansoni *is unclear.

### Tyrosine kinases

#### TK group

PTKs can be classified, based on the presence or absence of transmembrane domains, into receptor tyrosine kinase (RTK) that relay intracellular signals [74], and cytoplasmatic tyrosine kinase (CTK). *S. mansoni *kinome contains 15 RTKs and 19 CTKs. The 15 RTK include two InsRs (Insulin Receptors), four EGFRs (Epidermal Growth Factor Receptor), two VKRs (Venus Flytrap Kinase Receptors), a representative for Ephs (Ephrin receptors), Ror, CCK4 (Colon Carcinoma Kinase 4), and MUSK (Muscle_Specific kinase) families, besides three unknown receptors.

Two InsRs in *S. mansoni*, SmIR-1 (Smp_009990) and SmIR-2 (Smp_074030) present distinct functions during parasite development. These two receptors are well clustered within the InsR families but showed to be more divergent than the mammalian and *D. melanogaster *proteins (Additional file 7). SmIR-1 was localized in the muscles, intestinal epithelium, and basal membrane of adult male and female worms and at the periphery of schistosomula, mainly in the tegument [75]. SmIR-1 co-localized in schistosome tegument with glucose transporters suggesting a role in the regulation of glucose uptake which is an essential nutrient for the intra-mammalian stages of *S. mansoni*. SmIR-2, in contrast, was distributed in the parenchyma of adult males and females indicating a possible involvement of the receptor in parasite growth. *S. mansoni *is the first invertebrate with two insulin receptors characterized that seem to have distinct functions, as in vertebrates [18,75,76]. Mammals have two InsR members; insulin-like growth factor receptor (IGFR), which has a role in controlling growth, and (InR) which has specialized in metabolic regulation [77].

In *C. elegans *EGFR signaling induces behavioral quiescence [78]. One *S. mansoni *EGFR homolog (Smp_173590) was localized in the parasite muscle and perhaps related to muscle development or function [79]. Vertebrate EGF activates *S. mansoni *EGFR and the downstream classical ERK pathway (Figure 3), indicating the conservation of EGFR function in *S. mansoni *[79]. Moreover, human EGF was shown to increase protein and DNA synthesis as well as protein phosphorylation in parasites, supporting the hypothesis that host EGF could regulate schistosome development [80]. The similarity of schistosome proteins to sex hormone receptors of mammalian hosts provides a good example of host parasite relationship, where the adult worm depends on the host hormone synthesis for their maturation and reproduction [3].

Five *S. mansoni *proteins are not clustered with the main RTK families as shown in our phylogenetic analyses (Additional file 7). Three of them have a truncated catalytic domain (Smp_175590, Smp_093500 and Smp_157300) and two are specific RTK with a venus flytrap domain (VKR family). VKR is a family of receptors found in invertebrates, especially in insects. One *S. mansoni *VKR protein, Smp_153500 (SmVKR), was recently studied [81]. We identified another protein (Smp_019790) clustering with SmVKR (Additional file 7) with a high similarity. Despite the similarity of the catalytic domain of VKR protein with the IRs, these two proteins are not clustered with InsR family. In this respect, the most interesting finding is that VKR family members are not found in mammals and could represent good targets for drug development as a specific inhibitor for this family will probably not affect any protein of the host [81].

The CTKs in *S. mansoni *are represented by 11 different families (Additional file 7). SmTK3 (Smp_054500) and SmTK5 (Smp_136300) - src family members, and SmTK4 (Smp_149460) - syk family, are present in reproductive organs and possibly involved in the development of gonads and multiplication of germinal and vitelline cells [82-84]. Abl proteins of *S. mansoni *(Smp_128790 and Smp_169230) were recently studied using a Abl specific inhibitor (Imatinib, Gleevac^®^). The results showed an important morphological alteration in adult worms of *S. mansoni *that led to the death of the parasites [21]. *C. elegans *contains 42 members of the Fer family, while only a single member, SmFes, was found in *S. mansoni*. The Fer gene of *S. mansoni *(SmFes, Smp_164810) exhibits the characteristic features of Fes/Fps/Fer (fes, feline sarcoma; fps, Fujinami poultry sarcoma; fer, fes related) PTKs. By immunolocalization assays it was shown that SmFes is particularly expressed at the terebratorium of miracidia and tegument of cercaria and schistosomula skin-stage. These findings suggest that SmFes may play a role in signal transduction pathways involved in larval transformation after penetration into intermediate and definitive hosts [85,86].

#### RGC group

Proteins in this group share sequence similarity to the catalytic domain (Pfam: PF07714) found in proteins of the TK group [87]. The RGC group is underrepresented in most species, except in *C. elegans *that has a large expansion of these proteins and *S. cerevisiae *that has no protein with similarity to the TK catalytic domain (Figure 2). Only three RGC members were identified in the *S. mansoni *ePKinome. All of them are more closely related to the mammalian and insect families than the worm family. *C. elegans *and *B. malayi *RGC proteins form at least two different families noticeably more divergent from *S. mansoni*, *D. melanogaster*, *M. musculus*, and *H. sapiens *families as suggested by our phylogenetic analysis (Additional file 8). Most RGC proteins remain functionally uncharacterized. In *C. elegans*, several RGC proteins are highly expressed in restricted sets of neurons and are implicated in chemosensation. One RGC is involved in dauer stage formation [88]. Other parasites such as *L. major, T. brucei, T. cruzi *and *P. falciparum *also lack homologs in the RGC group [20,89]. The three *S. mansoni *RGC proteins have an amino acid substitution in the aspartic acid in subdomain VIb of the catalytic domain, rendering them catalytically inactive. Although the catalytic center of an enzyme is usually highly conserved, there have been reports of proteins, like those of the RGC group of ePKs, with substitutions at essential catalytic positions, which convert the enzyme into a catalytically inactive form. A recent study showed that inactive enzymes are found in a large variety of families conserved among metazoan species and they have lost their catalytic activity, have adopted new functions, and are involved in regulatory processes [34,90].

### Hybrid protein kinase

#### TKL Group

TKL consists of a divergent group that is phylogenetically close to the tyrosine kinases (Figure 3). However, TKL proteins have an unusual catalytic domain that is a hybrid between the serine/threonine and tyrosine kinases [32]. The catalytic domain may display greater similarity to the tyrosine catalytic domain (Pfam: PF07714) or to the serine/threonine catalytic domains (Pfam: PF00069) [10,11]. In *S. mansoni*, the TKL group includes MLK (Miked Lineage Kinases), LISK (Family containing closely related LIMK and TESK sub-families), Raf, RIPK (Receptor Interacting Protein Kinase), STKR (Serine/threonine kinase Receptors for activin and TGFβ ligands), and LRRK (Leucine Rich Repeat Kinase) (type 1 and type 2) families. Of the 19 TKL proteins found in *S. mansoni*, 15 display greater similarity to the serine/threonine catalytic domain and four (of Raf and MKL/ILK families) to the tyrosine catalytic domain. *S. mansoni *has no homologous proteins of the IRAK (interleukin-1 (IL-1) receptor-associated kinase) family that is present in *C. elegans*, *B. malayi, D. melanogaster, Homo sapiens*, and *M. musculus *(Additional file 9). Although *S. cerevisiae *does not have any TKL protein homologue, other fungal species do contain such proteins [14]. Raf (also known as MAPKKK) is a TKL family that plays an important role in the activation of STE proteins in the signaling cascade that culminates in the activation of ERK1/2 (Figure 4) [63]. A recent study showed that blocking the expression of the homolog of the *S. mansoni *Raf protein (Smp_176990) in *C. elegans *by RNAi, generate a sterile phenotype, which supports the hypothesis of the involvement of Raf protein in the germline development, somatic gonad development, oogenesis, spermatogenesis, ovulation or fertilization (Table 2). Raf protein may represents a good target for drug development in *S. mansoni*.

A STKR member that binds to TGFβ (Transforming growth factor) is a membrane receptor that can be divided into two subclasses (Type I and Type II). The type II receptor binds TGFβ and then recruits the type I receptor. The TGFβ type I receptor was cloned in *S. mansoni *(SmTβRI - Smp_173400.2) and it was found to be localized in the parasite surface [91,92]. Other type I STRK (Smp_049760) was identified in the *S. mansoni *predicted proteome and was not experimentally characterized so far (Additional file 9). Three type II STKRs (TGFβ type II receptors) are proteins identified in the same contig which were predicted to be a product of alternative splicing. A recent study revealed the presence of two transcripts that are translated into two different isoforms of type II receptor [93]. These transcripts are produced from the same gene by alternative splicing of the last two exons. The authors indicated that these different type II receptors might signal in different cells or development stages. Furthermore, that study showed that in the presence of human TGFβ, SmTβRII (Smp_165310) activated SmTβRI. The results also provide evidence for the role for the TGF-β signaling pathway in male-induced female reproductive development [94,95].

#### Other Group

The Other group consists of a mixed collection of kinases with representatives in higher eukaryotes, including SCY1, NEK (Mitotic Kinase family, also known as NRK), PEK, Haspin, WEE, NAK (Numb-Associated Kinase), ULK (Unc-51 Like Kinase), IRE (Inositol Requiring), PLK (Polo Like Kinases), AUR (Aurora Kinase), and CDC7 (Cell Division Control 7) families (Additional file 1). Our analysis showed that 15% of the *S. mansoni *ePKinome do not fall into any of the eight major groups, but include 20 smaller and conserved families.

### Accessory Domains

**Figure 5:**
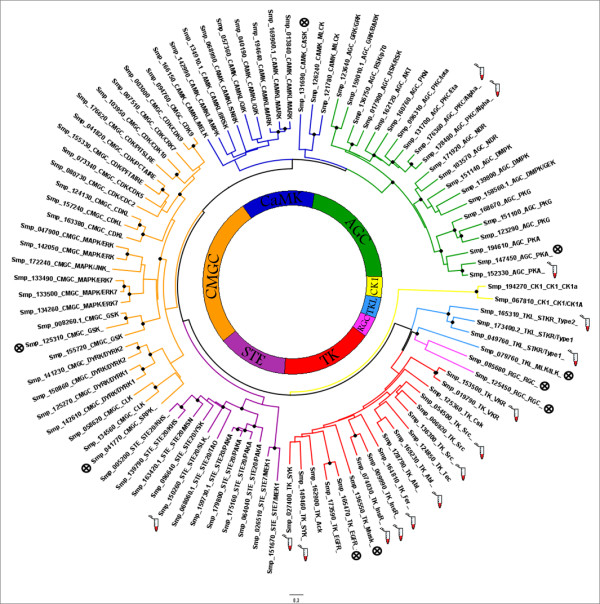
**Phylogenetic analysis of the paralogous ePKs groups of *S. Mansoni***. The catalytic domain of *S. mansoni *ePKs was used to construct a distance-based phylogenetic tree using PHYLIP programs. Some ePK were excluded from the tree after filtered the alighments to keep proteins with 30% to 98% pairwise sequence identity. The major ePK groups are color coded and include: CaMK (dark blue), CMGC (orange), TK (red), AGC (green), STE (pink), TKL (light blue), CK1 (yellow), and RGC (light pink). Functional classification is indicated individually in the protein. Proteins with experimental evidence () and those predicted to be inactive (⊗) due one or more substitutions in important residues in the catalytic domain are indicated. Bootstrap values (100 replicates) equal or higher than 80% are indicated (●).

The structure of the catalytic domain of many ePKs is highly conserved across distinct organisms because of the fact that all ePKs recognize and bind ATP at common sites. However, only the catalytic domain (architecture/sequence) is sufficiently divergent to enable the discrimination of groups, families, and subfamilies (Figure 5).

Most ePKs also have a second domain that is involved in protein-protein interaction and allosteric regulation of the catalytic domain [17]. In this work, only the catalytic domain sequence was used in the phylogenetic analyses. Interestingly, when the information on the ePK accessory domains was integrated into the phylogenies, we observed a correlation between diversity of protein architecture and the phylogenetic patterning. We also believe that the diversification of the ePKs happened a long time ago.

**Figure 6:**
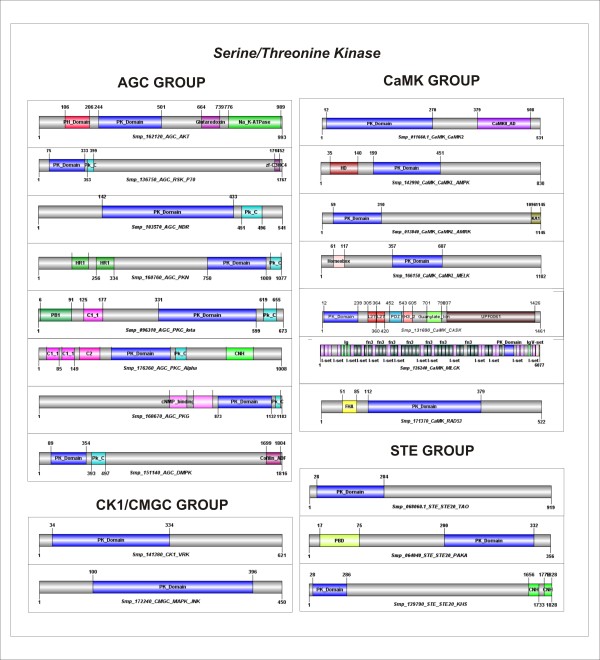
***S. mansoni *ePKs domain architectures**. Representative domain organizations of some *S. mansoni *ePKs belonging to the AGC, CaMK, CK1, CMGC, and STE groups are shown. Each protein ID and classification is shown below each image. Abbreviations followed are: PK_Domain (Protein kinase domain), PH_Domain (Pleckstrin Homology domain), Na_K_ATPase (Sodium/potassium ATPase beta chain), PK_C- (Protein Kinase C terminal domain), Zf-C3HC4 (zinc-finger, C3HC4 type RING finger), HR1 (Hr1 repeat), PB1 (Phox and Bem1p domain), C1_1 (Phorbol esters/diacylglycerol binding domain), C2 (Ca^2+^-dependent domain), CNH (citron homology domain), cNMP_binding (cyclic nucleotide-binding domain), Cofilin_ADF (Cofilin/tropomyosin-type actin-binding protein), CaMKII_AD (Calcium/calmodulin dependent protein kinase II Association), HD (HD homeobox domain), L27 (L27 domain), PDZ (PDZ domain, also known as DHR or GLGF), SH3_2 (Variant SH3 domain), Guanylate_kin (Guanylate kinase), UPF0061 (Uncharacterized ACR, YdiU/UPF0061 family), Ig (Immunoglobulin domain), fn3 (Fibronectin type III domain), V-set (Immunoglobulin V-set domain), FHA (Forkhead-associated domain), and PBD (P21-Rho-binding domain). The protein domain architectures were generated using DOG 1.0 [111] based on the Pfam domain limits [96].

**Figure 7:**
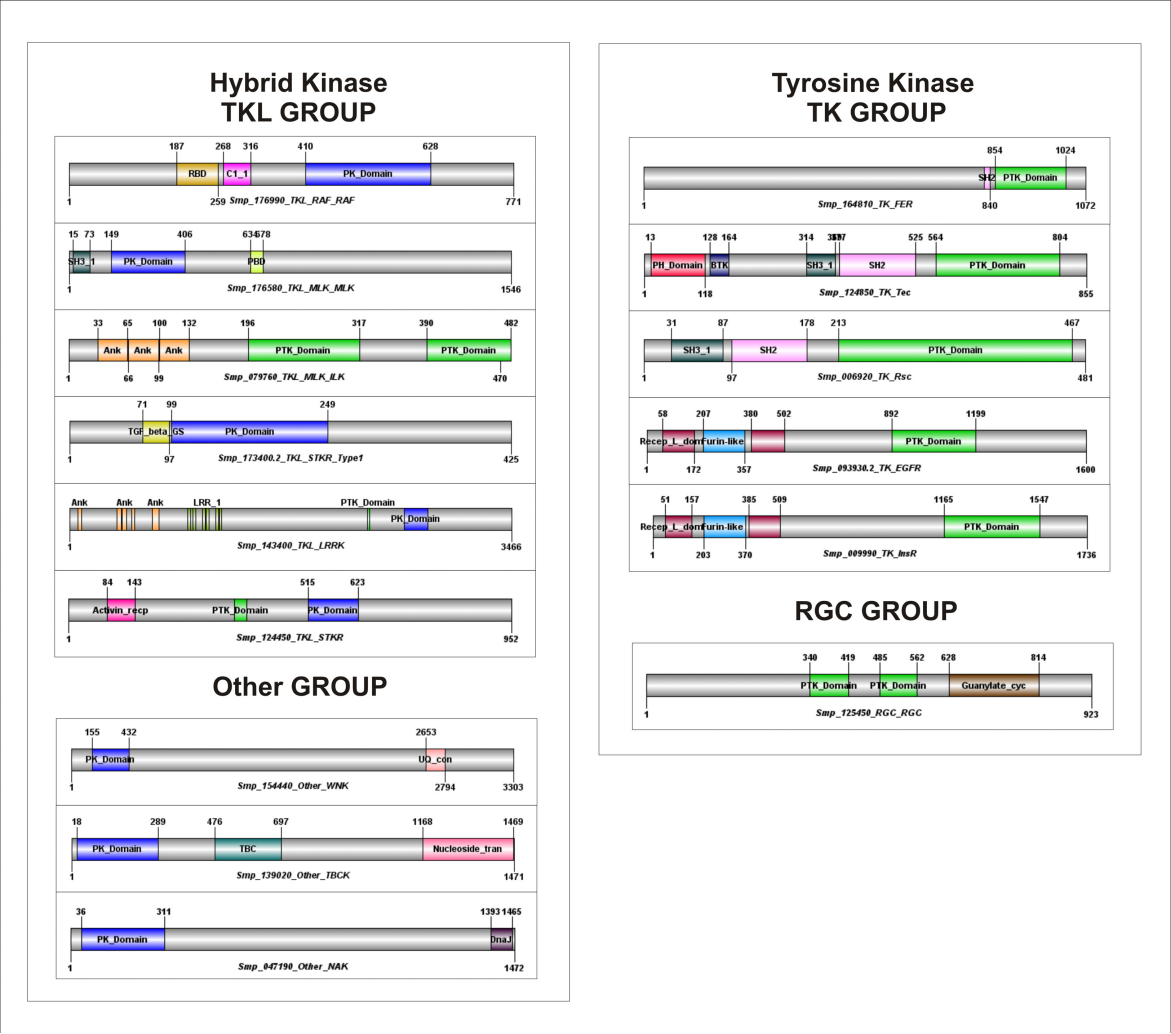
**Accessory Domains**. Representative domain organizations of some *S. mansoni *ePKs belonging to the TKL, Other, TK and, RGC groups are shown. Each protein ID and classification is shown below each image. Abbreviations followed are: PK_domain (Protein kinase domain), RBD (Raf-like Ras-binding domain), C1_1 (Phorbol esters/diacylglycerol binding domain), SH3_1 (Src homology 3 domain), PBD (P21-Rho-binding domain), Ank (Ankyrin repeat), PTK_domain (Protein tyrosine kinase domain), TGF_beta_GS (Transforming growth factor beta type I GS-motif), LRR_1 (Leucine Rich Repeat), Activin_recep (Activin types I and II receptor domain) UQ_con (Ubiquitin-conjugating enzyme) TBC (TBC domain), Nucleoside_tran (Nucleoside transporter) DnaJ (DnaJ domains), SH2 (Src homology 2 domain) PH_domain (Pleckstrin Homology domain) BTK (Bruton's tyrosine kinase motif) Recep_L_domain (Receptor L domain) furin-like (Furin-like cysteine rich region) Guanylate_cyc (Receptor family ligand binding region). The protein domain architectures were generated using DOG 1.0 [111] based on the Pfam domain limits [96].

The analysis of the sequence domain data from Pfam [96] showed that approximately 30% of *S. mansoni *ePKs are multi-domain proteins containing various regulatory and signaling domains tethered to catalytic kinase domains (Figures 6 and 7). It is known that the distinct protein architectures reflect functional differences among proteins [32]. Hence, understanding the mechanisms that generate such diverse repertoire of protein architectures is essential to the comprehension of the biological function of the ePKs. Furthermore, we observed in ePKs of *S. mansoni *some unusual architecture that probably occurs by domain fusion and recruitment (see some examples below), generating specificity towards cognate substrates and regulators in this parasite.

The most common Pfam accessory domains found in *S. mansoni *kinases are Pkinase_C (Pfam: PF00433) all found in the AGC group; C1_1 (Pfam: PF00130) found in the AGC and TKL groups; SH2 (PF00017) all found in the TK group; and SH3 (Pfam: PF00018) found in TK and TKL groups. These domains are commonly found in protein kinase families as we observed in other species from KinBase [15].

More than 40% of *S. mansoni *AGC group have the PKinase_C domain associated with the catalytic domain. The C1_1 domain is conserved in N-terminal regions of all PKC proteins of *S. mansoni *(Figure 6) and has been shown to bind PE (phorbol esters) and DAG (diacylglycerol). DAG is an important second messenger and Phorbol esters are analogues of DAG [32,96]. The C1_1 domain is present in one or two copies depending on the isozyme of PKC (Figure 6). cNMP_binding is a N-terminal domain of PKG proteins that bind cyclic nucleotides (cAMP or cGMP) to relieve the inhibition of the catalytic domain [15,35]. The AKT protein of *S. mansoni *(Smp_162120) has an unusual domain combination (Figure 6) as the two C-terminal domains (Glutaredoxin and Na_K-ATPase) are not found in *D. melanogaster, C. elegans, M. musculus *and *H. sapiens*.

CASK is a member of the CaMK group and plays a key role in establishing inter-cellular contacts and plasticity at cellular junctions [54]. The accessory domains found in *S. mansoni *CASK protein (L27 and PDZ domains, which serve as protein interaction modules, SH3, and a C-terminal guanylate kinase domains) are conserved in higher eukaryotes. However, the UPF0061 (Pfam: PF02696) is uncharacterized [96] and possesses an unusual domain found in the C-terminal region of *S. mansoni *CASK protein (Figure 6). The long protein kinase MLCK (Figure 5a) possesses a large number of Ig repeats (I-set, V-set and Ig) that, in other species, are involved in a variety of functions, including cell-cell recognition, cell-surface receptors, muscle structure and the immune system [96], and fn3 repeats, that is an approximately 100 amino acid domain commonly found in a variety of organisms.

The CMGC and CK1 groups have none or a few accessory domains in *S. mansoni*. However, it is known that small regions in these proteins play an important role in recognizing and binding to the substrate [97,98]. For example, the CD domain (common docking domain) is a C-terminal region of MAPK proteins composed of a set of negatively charged amino acids that is used to anchor protein activators (such as STE proteins), substrates (such as MAPKAPK) and inactivating proteins (such as MAPK phosphatases) [99]. Thus, this region governs a series of signal transduction in the cascade of reactions of MAPKs. Other regions, including the ED (ERK docking) site, working with the CD domain and ensuring specificity and interaction strength [99].

PBD (p21-Rho-binding) and C-terminal CNH domain are usually found in the STE20 families (Figure 6). PBD binds to cdc42-GTPases activating the signaling cascade which act upstream in the MAPK cascade. The CNH domain interacts with the small GTPase and regulating the actin cytoskeleton [96].

The SH3 and SH2 (Pfam: PF00018 and PF00017, respectively) domains are common found in CTK proteins. SH2 function as regulatory modules of intracellular signaling cascades and it was found in eight out of 19 *S. mansoni *CTKs. Fer PTK is usually composed of three domains, FHC domain, SH2, and C-terminal kinase domain as it occurs in Fer proteins of *H. sapiens, M. musculus*, and *D. melanogaster*. However, the *S. mansoni *Fer protein (SmFes - Smp_164810) [86] and the 42 Fer proteins of *C. elegans *seems to have lost the N-terminal FHC domain (Figure 7). RTKs are characterized by an extracellular domains, a membrane spanning segment and an intracellular kinase domain [32]. The extracellular ligand binding domain of EGFR and InsR proteins are composed of two receptor_L sandwiching a Furin_like domain (Figure 7). SmVKR is composed of an unusual extracellular Venus flytrap module (VFT) linked through a single transmembrane domain to an intracellular tyrosine catalytic domain similar to that of the insulin receptor and a putative function in reproduction and development was observed [81]. Other extracellular domains found in *S. mansoni *are Ephrin_Ibd (Pfam: PF01404) in the Ephrin recptors (Eph) and Ig domains (Pfam: PF00047) in CCK4 proteins (Additional file 1).

In conclusion, the protein architecture, including the accessory domains, may indicate potential protein partners. Signaling roles of schistosome specificities or unusual architectures are of special biological interest.

## Conclusions

This study allowed us to identify and classify 252 ePKs encoded in the predicted proteome of *S. mansoni*. Together, these proteins represent 1.9% of the proteome and indicate that protein phosphorylation is an important mechanism for regulating the complex life cycle of the parasite. We improve the functional annotation of 40% of *S. mansoni *ePKs (Table 3) by applying a phylogenetic framework. Moreover, it was possible to gain insights into kinase function once 94% of the *S. mansoni *ePKinome had previously an unknown function. *S. mansoni *has proteins in each ePKs group. Most of them are clearly clustered with known kinases from other eukaryotes with no family being exclusively found or expanded in *S. mansoni*. Some proteins are not clustered with the main ePK family as the catalytic domain is truncate, indicating that the current gene/protein predictions require further refinement. Proteins were mentioned as potential targets for drug design and development as they may play an essential function in the parasite. Furthermore new and effective drugs bind PKs close but not in the ATP site and occlude ATP access to the kinase to retard enzyme activity [17]. So, proteins of *S. mansoni *with a sequence highly similar to host proteins can be used as protein targets since the inhibitor binds in non-conserved residues outside the ATP site. Also, the unusual domains found in *S. mansoni *can be used for constructing more specific *S. mansoni *inhibitors. Moreover, as we continue this work, we will highlight the biochemical and physiological adaptations of *S. mansoni *in response to diverse environments during parasite development, vector interaction, and host infection.

**Table 3:** *S. mansoni *ePKinome annotation improved by phylogenomics

After phylogenetic analysis		
**Modifications**	**Number of changes**	**% of *S. mansoni *ePKinome**

Change group classification	14	5,55%
Change family classification	18	7,14%
Add subfamily	61	24,20%
Classification of unknown proteins	11	3,96%

**Total of changes**	**104**	**40,85%**

Through phylogenetic analysis, we have improved the functional annotation of 40% of *S. mansoni *ePKs by changing protein classification at the group and family levels, assigning subfamilies, and classifying proteins without any previous classification.

## Methods

### Organisms and Sequences

*S. mansoni *[NCBI taxid: 6183] and six other organisms were selected for this work including *Homo sapiens *[taxid: 9606], *Mus musculus *[taxid: 10090], *Drosophila melanogaster *[taxid: 7227], *Caenorhabditis elegans *[taxid: 6239], *Brugia malayi *[taxid: 6279], and *Saccharomyces cerevisiae *[taxid: 4932]. The *S. mansoni *predicted proteome data was downloaded from *SchistoDB*, version [2.0] [29], which contains the original gene and genomic information provided by the Wellcome Trust Institute and described elsewhere [26]. Datasets of protein kinases from the other organisms were downloaded from the kinase database at Sugen/Salk - KinBase [15], except for *Brugia malayi*, which was retrieved from KEGG [100].

### Functional Classification

Functional classification of protein kinases into groups, families, and subfamilies followed the proposed hierarchy described elsewhere [12,13,32]. Potential protein kinases of *S. mansoni *were identified and characterized by combined approaches based on sequence similarity and phylogenetic relationships. These proteins were first identified by similarity to Hidden Markov Models (HMMs) as described below. Also based on sequence similarity, each predicted protein kinase was manually annotated by integrating data from InterProScan [101] and reverse PSI-BLAST (rpsblast) [102] output searches into Artemis [103]. Further analysis was performed by HMMs searching for non-catalytic (accessory) domains associated to the conserved catalytic domain of protein kinases based on data available at the Protein families database - Pfam [96]. Functional classification was also devised based on the literature and on the assumption of a broad conservation of the molecular functions. Phylogenetic analyses of the ePK kinases groups performed in the present work corroborated this classification as well as supported new functional assignments for previously uncharacterized proteins (see below).

### Hidden Markov Models

In order to identify potential homologs in *S. mansoni*, amino acid sequences of known protein kinases of five model organisms (*H. sapiens*, *M. musculus*, *D. melanogaster*, *C. elegans*, and *S. cerevisiae*) were selected. A total of 68 diverse amino acid sequences corresponding to the kinase catalytic domain and sharing less than 50% sequence identity were aligned in MAFFT [30] and manually-edited for further analysis. Local and global HMMs were built with the HMMer package http://hmmer.janelia.org from multiple sequence alignments and used for sensitive searches against the *S. mansoni *proteome [26].

### Phylogenetic Analyses

Amino acid sequences corresponding to the conserved catalytic domain (Pfam: PF0069 or PF07714) of each group of protein kinases were separately aligned using the default parameters of MAFFT [30]. Multiple sequence alignments were filtered to keep proteins sharing 50% to 90% pairwise sequence identity using the decreased redundancy tool [104] and manually edited to remove ambiguous regions using BioEdit [105]. Final alignments were used in phylogenetic reconstructions through multiple programs available in the Phylogeny Inference Package - PHYLIP, version 3.69 [31]. Initially, 1000 random datasets (replicates) were created for each alignment using *seqboot *with default parameters. For each dataset, it was calculated a distance matrix under the JTT model with gamma-distributed sites by *protdist*. Next, phylogenies were estimated from distance matrix data adopting the *Fitch-Margoliash *criterion as implemented in *fitch*. Finally, the results from the random datasets were summarized by *consense*, which computes consensus trees by the majority-rule consensus tree method. Phylogenetic trees were visualized and edited using the Tree Figure Drawing Tool - *FigTree*, version 1.3.1 [106]. Nodes with at least 80% bootstrap values were considered to support functional prediction.

## Abbreviations

ATP: adenosine triphosphate; cAMP: cyclic adenosine monophosphate; GTP: guanosine triphosphate; NCBI: National Center for Biotechnology Information; RNAi: RNA interference.

## Authors' contributions

LFA: carried out the functional annotation and phylogenetic studies, and drafted the manuscript. LAN: designed the phylogenetic studies, performed preliminary phylogenetic studies, coordinated the functional prediction, and co-wrote this manuscript. LGAV and LLS carried out the functional annotation based on sequence similarity search using HMMs. AZ wrote the Perl scripts for data manipulation and provided computational support for this study. JCR designed and performed the HMMs analysis. GO participated in the design and coordination of this study, and co-wrote the manuscript. All authors read and approved the final manuscript.

## Supplementary Material

Additional file 1 **Eukaryotic protein kinases of *Schistosoma mansoni***. Click here for file

Additional file 2 **Phylogenetic analysis of the AGC group**. Amino acid sequences of the catalytic domain of the AGC proteins of *S. mansoni *(blue labels), *C. elegans*, *D. melanogaster*, *S. cerevisiae*, *H. sapiens*, and *B. malayi *were used to construct a distance-based phylogenetic tree using PHYLIP programs. Bootstrap values (1000 replicates) equal or higher than 80% are indicated (●) as cutoff values to support the family/subfamily classification. Functional classification is indicated individually in the protein labels. Protein families are highlighted by the vertical bars and include: RSK (Ribosomal S6 Kinase), PKA (Protein Kinase A), PKG (Protein Kinase G), PDK1(phosphoinositide-dependent protein kinase 1), NDR (nuclear Dbf2-related kinases), DMPK (Myotonic Dystrophy Protein Kinase), MAST (Microtubule Associated Serine/Threonine Kinase), RSKL (RSK-like); PKC (Protein Kinase C), PKN (Protein Kinase N), Akt (also known as PKB, Protein Kinase B), SGK (Serum and Glucocorticoid Resposive Kinase), GRK (G-protein coupled Receptor Kinase), YANK (Yet Another Novel Kinase). Click here for file

Additional file 3 **Distribution of some ePKs Families in *S. mansoni *and model organisms**. *S. mansoni *proteins were classified according to KinBase [15] by combining sequence similarity searches (HMMs) and phylogenetic analysis (this work). For comparison, occurrence of the ePKs families in *C. elegans*, *D. melanogaster*, *S. cerevisiae*, and *H. sapiens *is shown. Click here for file

Additional file 4 **Phylogenetic analysis of the CaMK group**. Amino acid sequences of the catalytic domain of the CaMK proteins of *S. mansoni *(blue labels), *C. elegans*, *D. melanogaster*, *S. cerevisiae*, *H. sapiens*, and *B. malayi *were used to construct a distance-based phylogenetic tree using PHYLIP programs. Bootstrap values (1000 replicates) equal or higher than 80% are indicated (●) as cutoff values to support the family/subfamily classification. Functional classification is indicated individually in the protein labels. Protein families are highlighted by the vertical bars and include: MAPKAPK (MAP Kinase Associated Protein Kinase), CAMK1 (CAMK family 1), PHK (Phosphorylase Kinase), Trio, DAPK (Death Associated Protein Kinase), MLCK (Myosin Light Chain Kinases), CASK, CAMK2 (CAMK family 2), DCAMKL (Doublecortin and CaMK-Like), PKD (Protein Kinase D), RAD53, Trbl, CAMKL (CAMKL-like). Click here for file

Additional file 5 **Phylogenetic analysis of the CMGC group**. Amino acid sequences of the catalytic domain of the CMGC proteins of *S. mansoni *(blue labels), *C. elegans*, *D. melanogaster*, *S. cerevisiae*, *H. sapiens*, and *B. malayi *were used to construct a distance-based phylogenetic tree using PHYLIP programs. Bootstrap values (1000 replicates) equal or higher than 80% are indicated (●) as cutoff values to support the family/subfamily classification. Functional classification is indicated individually in the protein labels. Protein families are highlighted by the vertical bars and include: GSK (Gycogen Synthase 3 Kinase), MAPK (Mitogen Activated Protein Kinase), CK2 (Cell Kinase 2), CDKL (Cyclin Dependent Kinase Like), CDK (Cyclin Dependent Kinase), SRPK (SR Protein Kinase; phosphorylates SR splicing factors), DYRK (Dual-specificity Y (tyrosine) Regulated Kinase), CLK (CDC-Like Kinase). Click here for file

Additional file 6 **Phylogenetic analysis of the STE group**. Amino acid sequences of the catalytic domain of the STE proteins of *S. mansoni *(blue labels), *C. elegans*, *D. melanogaster*, *S. cerevisiae*, *H. sapiens*, and *B. malayi *were used to construct a distance-based phylogenetic tree using PHYLIP programs. Bootstrap values (1000 replicates) equal or higher than 80% are indicated (●) as cutoff values to support the family/subfamily classification. Functional classification is indicated individually in the protein labels. Protein families are highlighted by the vertical bars and include: STE11 (MAP3K (MAP kinase kinase kinase) genes), STE20 (MAP4K (MAP kinase kinase kinase kinase) genes), STE7 (MAP2K (MAP kinase kinase) genes). Click here for file

Additional file 7 **Phylogenetic analysis of the TK group**. Amino acid sequences of the catalytic domain of the TK proteins of *S. mansoni *(blue labels), *C. elegans*, *D. melanogaster*, *S. cerevisiae*, *H. sapiens*, and *B. malayi *were used to construct a distance-based phylogenetic tree using PHYLIP programs. Bootstrap values (1000 replicates) equal or higher than 80% are indicated (●) as cutoff values to support the family/subfamily classification. Functional classification is indicated individually in the protein labels. Protein families are highlighted by the vertical bars and include: JAK, Ack (Activated Cdc42-associated tyrosine kinase), Syk, FAK (Focal Adhesion Kinase), VKR, Fer, Sev, ALK (Anaplastic Lymphoma Kinase), InsR (Insulin Receptor), DDR (Discoidin Domain Receptor kinase), Musk (Muscle-Specific Kinase), Ror, Lmr (Lemur Kinase), Eph (Ephrin receptors), Axl (Also known as TAM (Tyro3, Axl, Mer)), Ryk, EGFR (Epidermal Growth Factor Receptor), FGFR (Fibroblast Growth Factor Receptor), PDGFR, VEGFR, Kin16, Jak (Janus Kinases), Tec, Abl (Abelson murine leukemia homolog), and Src. Click here for file

Additional file 8 **Phylogenetic analysis of the RGC group**. Amino acid sequences of the catalytic domain of the RGC proteins of *S. mansoni *(blue labels), *C. elegans*, *D. melanogaster*, *S. cerevisiae*, *H. sapiens*, and *B. malayi *were used to construct a distance-based phylogenetic tree using PHYLIP programs. Bootstrap values (1000 replicates) equal or higher than 80% are indicated (●) as cutoff values to support the family/subfamily classification. Functional classification is indicated individually in the protein labels. Click here for file

Additional file 9 **Phylogenetic analysis of the TKL group**. Amino acid sequences of the catalytic domain of the CK1 proteins of *S. mansoni *(blue labels), *C. elegans*, *D. melanogaster*, *S. cerevisiae*, *H. sapiens*, and *B. malayi *were used to construct a distance-based phylogenetic tree using PHYLIP programs. Bootstrap values (1000 replicates) equal or higher than 80% are indicated (●) as cutoff values to support the family/subfamily classification. Functional classification is indicated individually in the protein labels. Protein families are highlighted by the vertical bars and include: IRAK (IL1 Receptor Associated Kinase), RIPK (Receptor Interacting Protein Kinases), LISK (Family containing closely related TESK and LIMK sub-families), MLK (Miked Lineage Kinases), LRRK (Leucine Rich Repeat Kinase), RAF, STKR (Serine/Threonine Kinase Receptors - receptors for activin and TGFb ligands). Click here for file
