# Supplementary figures and images for: Eukaryotic Protein Kinases (ePKs) of the Helminth Parasite Schistosoma mansoni
Source: BMC Genomics. 2011 May 6;12:215. doi: 10.1186/1471-2164-12-215 (PMC3117856; doi:10.1186/1471-2164-12-215)

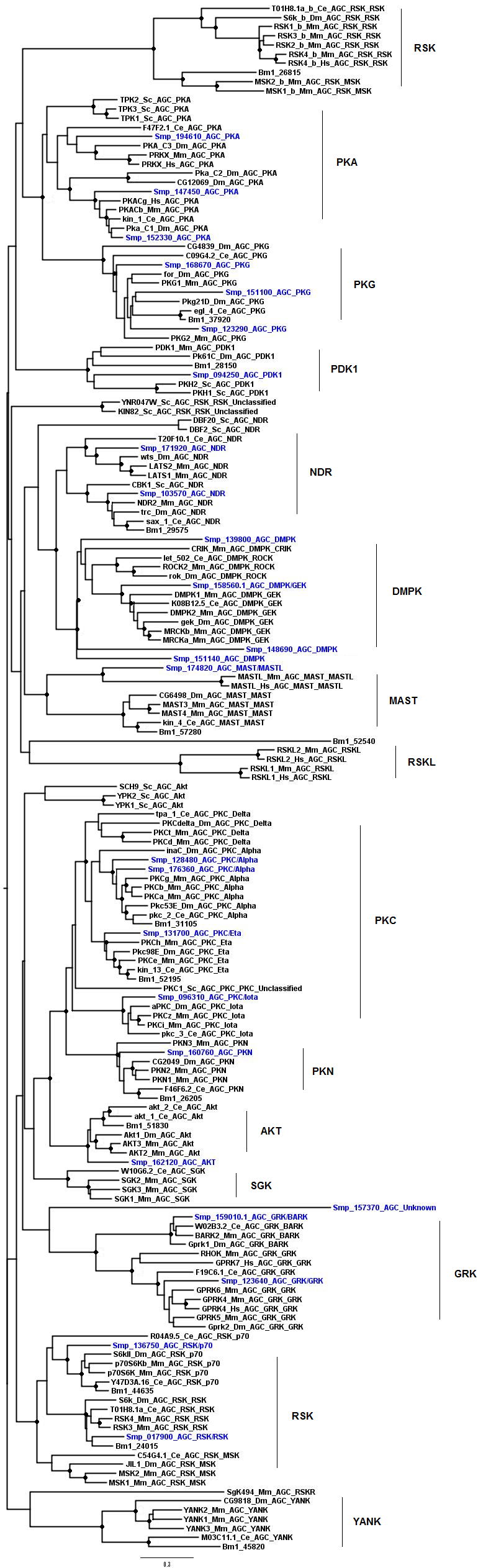

Supplement: Additional file 2 — Phylogenetic analysis of the AGC group. Amino acid sequences of the catalytic domain of the AGC proteins of S. mansoni (blue labels), C. elegans, D. melanogaster, S. cerevisiae, H. sapiens, and B. malayi were used to construct a distance-based phylogenetic tree using PHYLIP programs. Bootstrap values (1000 replicates) equal or higher than 80% are indicated (●) as cutoff values to support the family/subfamily classification. Functional classification is indicated individually in the protein labels. Protein families are highlighted by the vertical bars and include: RSK (Ribosomal S6 Kinase), PKA (Protein Kinase A), PKG (Protein Kinase G), PDK1(phosphoinositide-dependent protein kinase 1), NDR (nuclear Dbf2-related kinases), DMPK (Myotonic Dystrophy Protein Kinase), MAST (Microtubule Associated Serine/Threonine Kinase), RSKL (RSK-like); PKC (Protein Kinase C), PKN (Protein Kinase N), Akt (also known as PKB, Protein Kinase B), SGK (Serum and Glucocorticoid Resposive Kinase), GRK (G-protein coupled Receptor Kinase), YANK (Yet Another Novel Kinase). [file 1471-2164-12-215-S2.PNG]

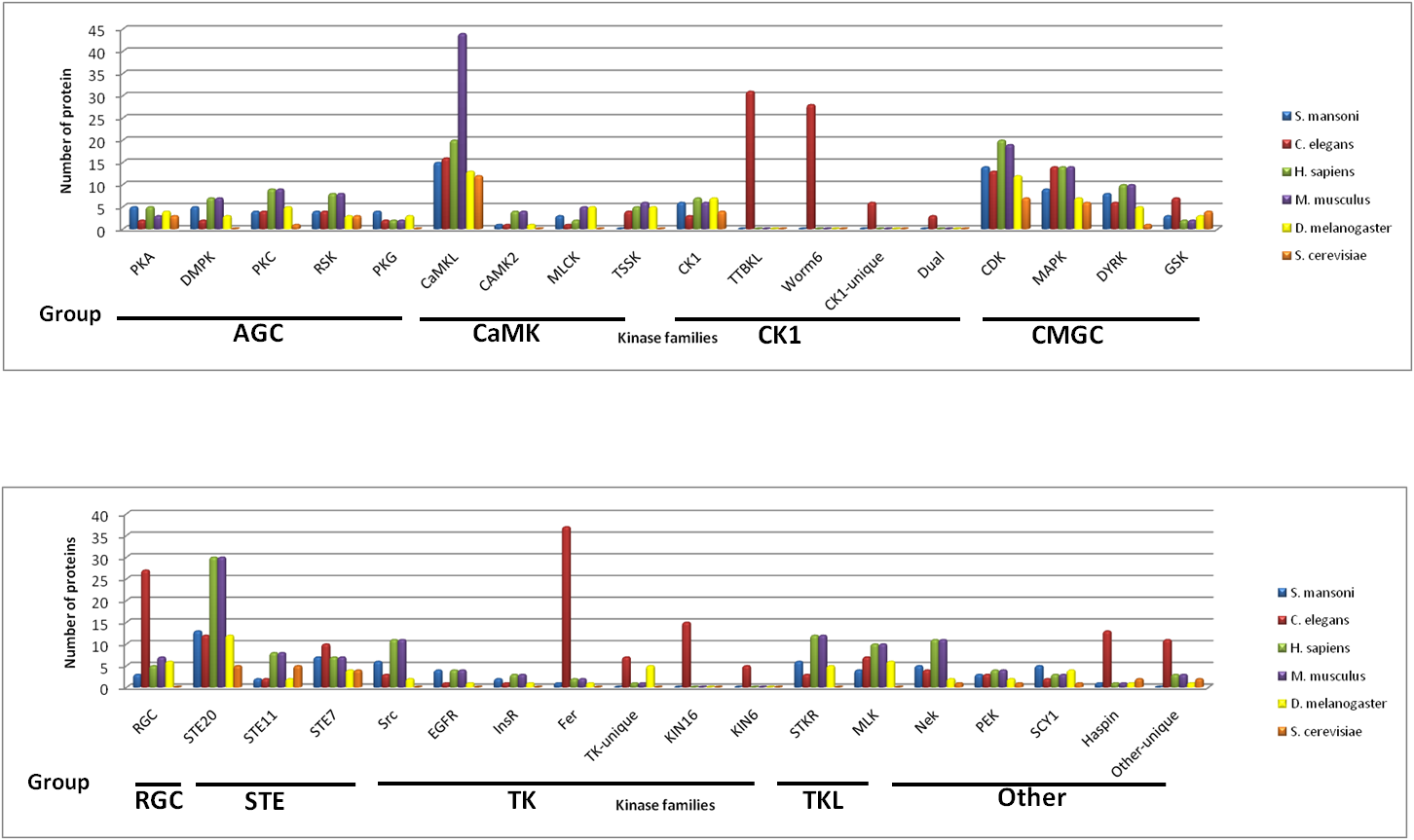

Supplement: Additional file 3 — Distribution of some ePKs Families in S. mansoni and model organisms. S. mansoni proteins were classified according to KinBase [15] by combining sequence similarity searches (HMMs) and phylogenetic analysis (this work). For comparison, occurrence of the ePKs families in C. elegans, D. melanogaster, S. cerevisiae, and H. sapiens is shown. [file 1471-2164-12-215-S3.PNG]

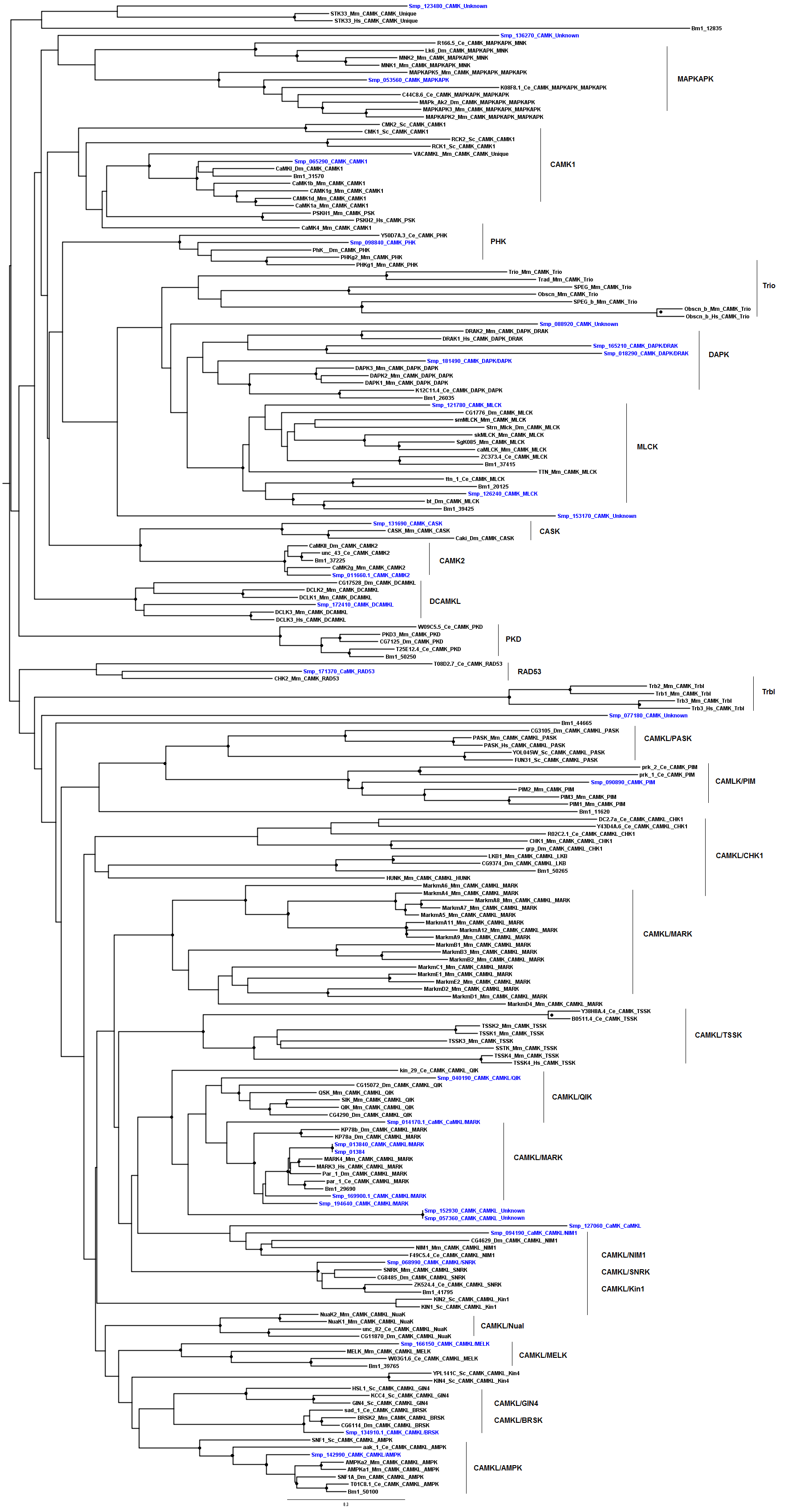

Supplement: Additional file 4 — Phylogenetic analysis of the CaMK group. Amino acid sequences of the catalytic domain of the CaMK proteins of S. mansoni (blue labels), C. elegans, D. melanogaster, S. cerevisiae, H. sapiens, and B. malayi were used to construct a distance-based phylogenetic tree using PHYLIP programs. Bootstrap values (1000 replicates) equal or higher than 80% are indicated (●) as cutoff values to support the family/subfamily classification. Functional classification is indicated individually in the protein labels. Protein families are highlighted by the vertical bars and include: MAPKAPK (MAP Kinase Associated Protein Kinase), CAMK1 (CAMK family 1), PHK (Phosphorylase Kinase), Trio, DAPK (Death Associated Protein Kinase), MLCK (Myosin Light Chain Kinases), CASK, CAMK2 (CAMK family 2), DCAMKL (Doublecortin and CaMK-Like), PKD (Protein Kinase D), RAD53, Trbl, CAMKL (CAMKL-like). [file 1471-2164-12-215-S4.PNG]

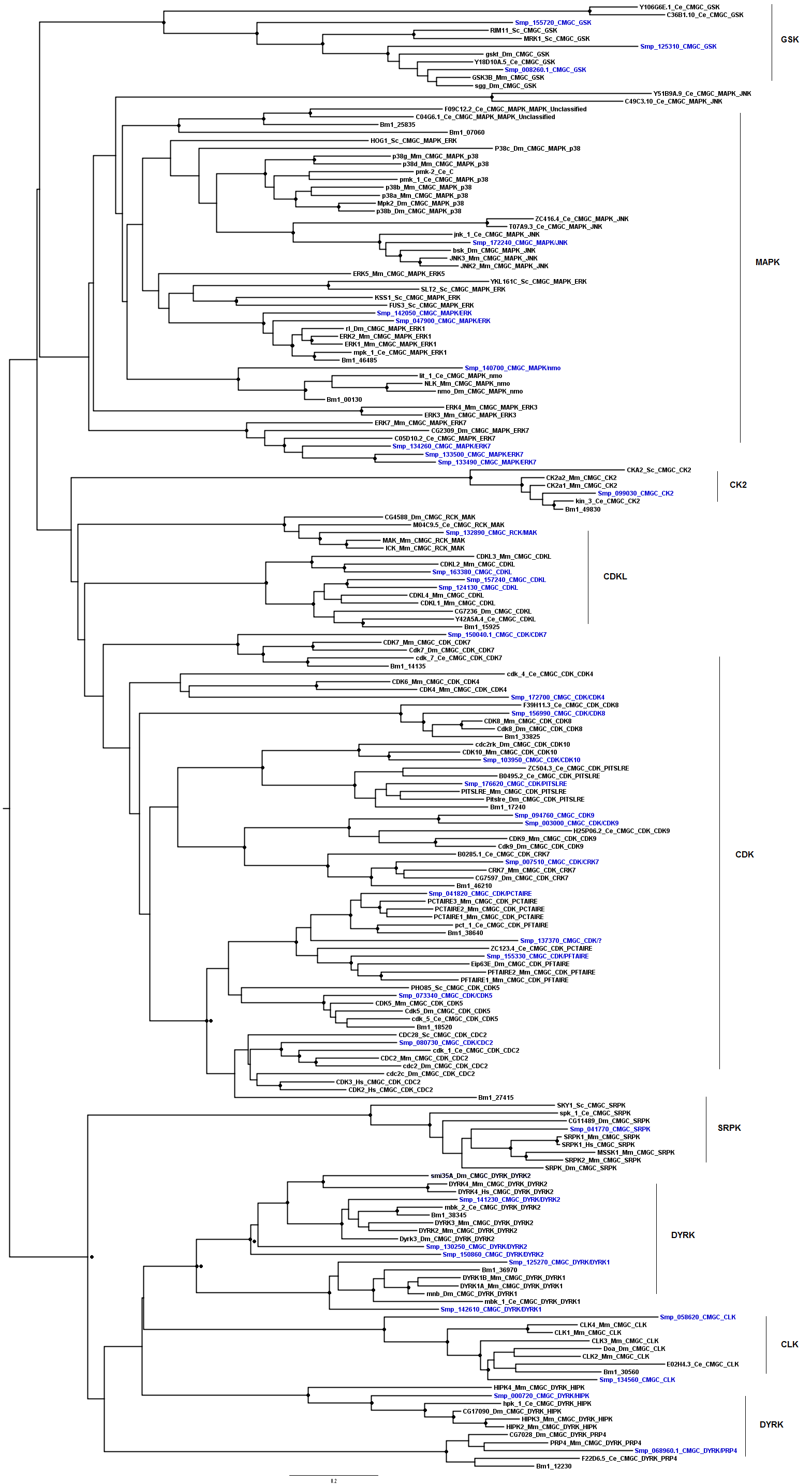

Supplement: Additional file 5 — Phylogenetic analysis of the CMGC group. Amino acid sequences of the catalytic domain of the CMGC proteins of S. mansoni (blue labels), C. elegans, D. melanogaster, S. cerevisiae, H. sapiens, and B. malayi were used to construct a distance-based phylogenetic tree using PHYLIP programs. Bootstrap values (1000 replicates) equal or higher than 80% are indicated (●) as cutoff values to support the family/subfamily classification. Functional classification is indicated individually in the protein labels. Protein families are highlighted by the vertical bars and include: GSK (Gycogen Synthase 3 Kinase), MAPK (Mitogen Activated Protein Kinase), CK2 (Cell Kinase 2), CDKL (Cyclin Dependent Kinase Like), CDK (Cyclin Dependent Kinase), SRPK (SR Protein Kinase; phosphorylates SR splicing factors), DYRK (Dual-specificity Y (tyrosine) Regulated Kinase), CLK (CDC-Like Kinase). [file 1471-2164-12-215-S5.PNG]

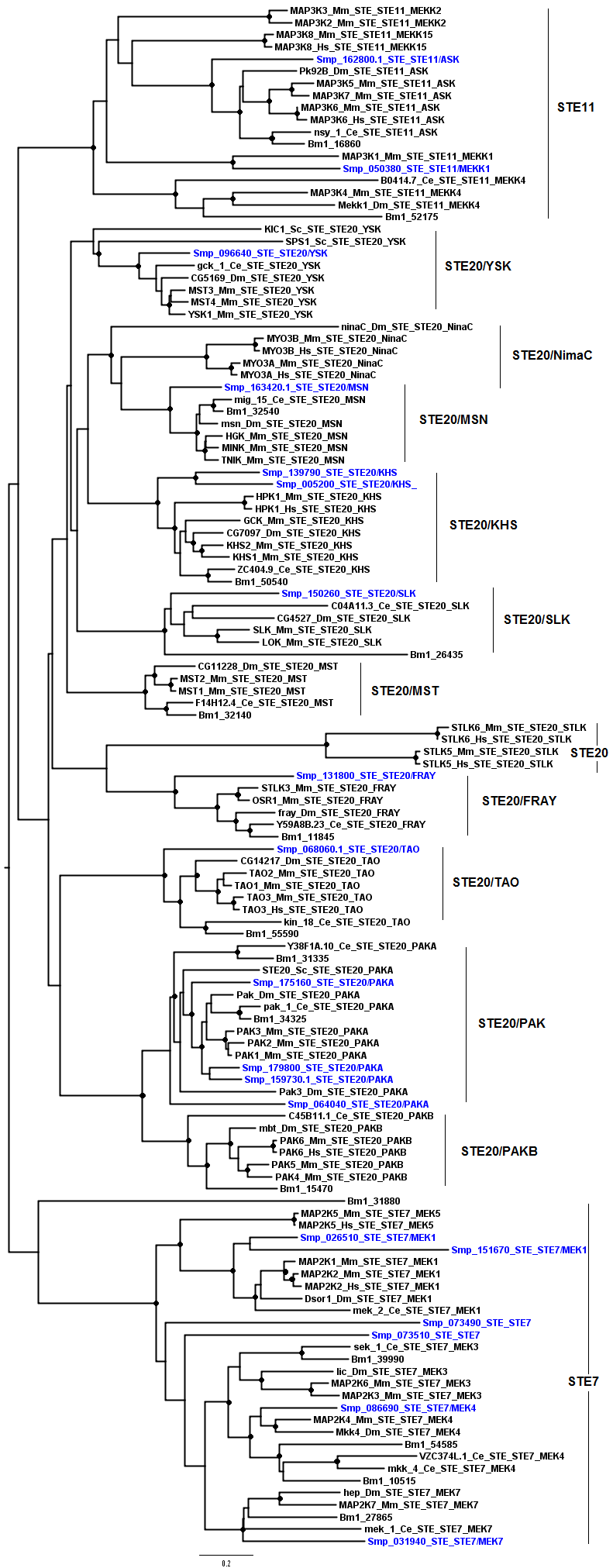

Supplement: Additional file 6 — Phylogenetic analysis of the STE group. Amino acid sequences of the catalytic domain of the STE proteins of S. mansoni (blue labels), C. elegans, D. melanogaster, S. cerevisiae, H. sapiens, and B. malayi were used to construct a distance-based phylogenetic tree using PHYLIP programs. Bootstrap values (1000 replicates) equal or higher than 80% are indicated (●) as cutoff values to support the family/subfamily classification. Functional classification is indicated individually in the protein labels. Protein families are highlighted by the vertical bars and include: STE11 (MAP3K (MAP kinase kinase kinase) genes), STE20 (MAP4K (MAP kinase kinase kinase kinase) genes), STE7 (MAP2K (MAP kinase kinase) genes). [file 1471-2164-12-215-S6.PNG]

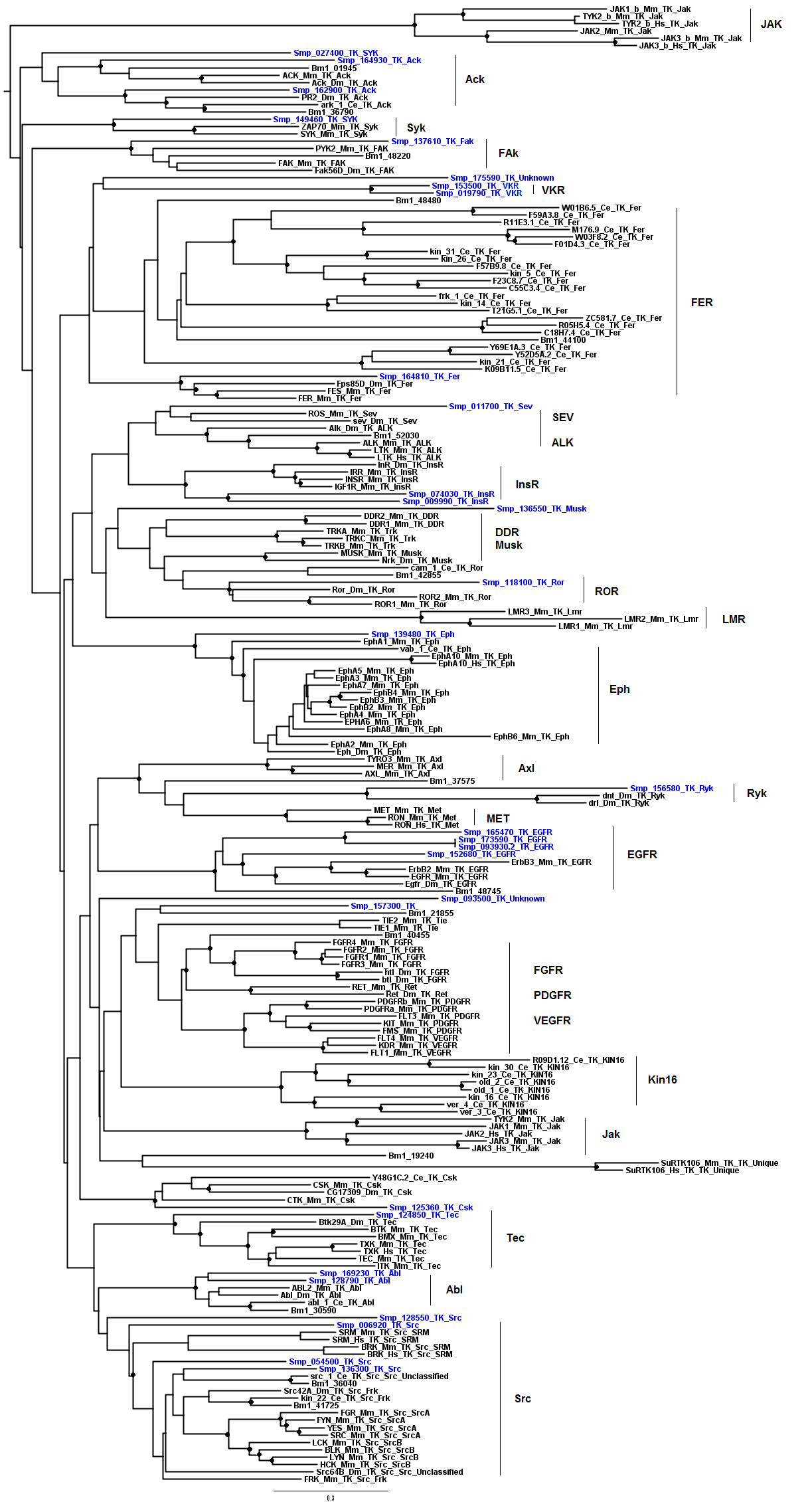

Supplement: Additional file 7 — Phylogenetic analysis of the TK group. Amino acid sequences of the catalytic domain of the TK proteins of S. mansoni (blue labels), C. elegans, D. melanogaster, S. cerevisiae, H. sapiens, and B. malayi were used to construct a distance-based phylogenetic tree using PHYLIP programs. Bootstrap values (1000 replicates) equal or higher than 80% are indicated (●) as cutoff values to support the family/subfamily classification. Functional classification is indicated individually in the protein labels. Protein families are highlighted by the vertical bars and include: JAK, Ack (Activated Cdc42-associated tyrosine kinase), Syk, FAK (Focal Adhesion Kinase), VKR, Fer, Sev, ALK (Anaplastic Lymphoma Kinase), InsR (Insulin Receptor), DDR (Discoidin Domain Receptor kinase), Musk (Muscle-Specific Kinase), Ror, Lmr (Lemur Kinase), Eph (Ephrin receptors), Axl (Also known as TAM (Tyro3, Axl, Mer)), Ryk, EGFR (Epidermal Growth Factor Receptor), FGFR (Fibroblast Growth Factor Receptor), PDGFR, VEGFR, Kin16, Jak (Janus Kinases), Tec, Abl (Abelson murine leukemia homolog), and Src. [file 1471-2164-12-215-S7.PNG]

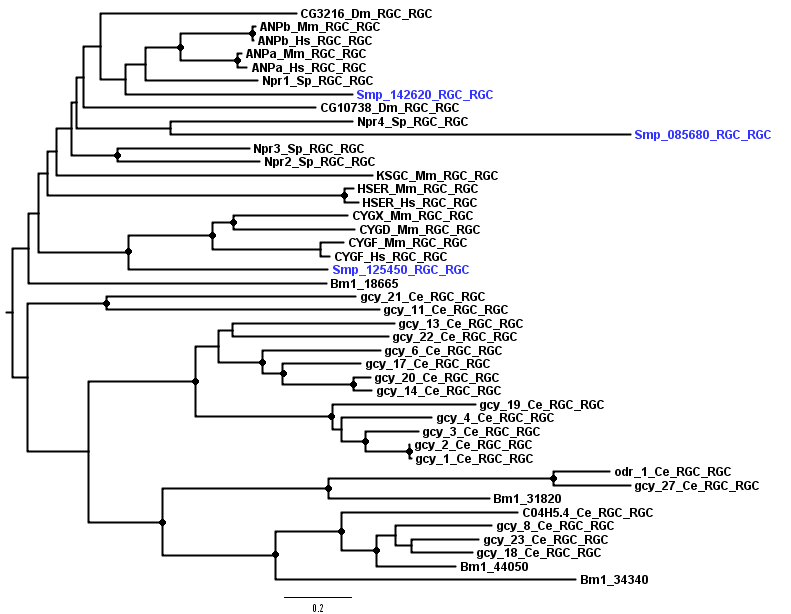

Supplement: Additional file 8 — Phylogenetic analysis of the RGC group. Amino acid sequences of the catalytic domain of the RGC proteins of S. mansoni (blue labels), C. elegans, D. melanogaster, S. cerevisiae, H. sapiens, and B. malayi were used to construct a distance-based phylogenetic tree using PHYLIP programs. Bootstrap values (1000 replicates) equal or higher than 80% are indicated (●) as cutoff values to support the family/subfamily classification. Functional classification is indicated individually in the protein labels. [file 1471-2164-12-215-S8.PNG]

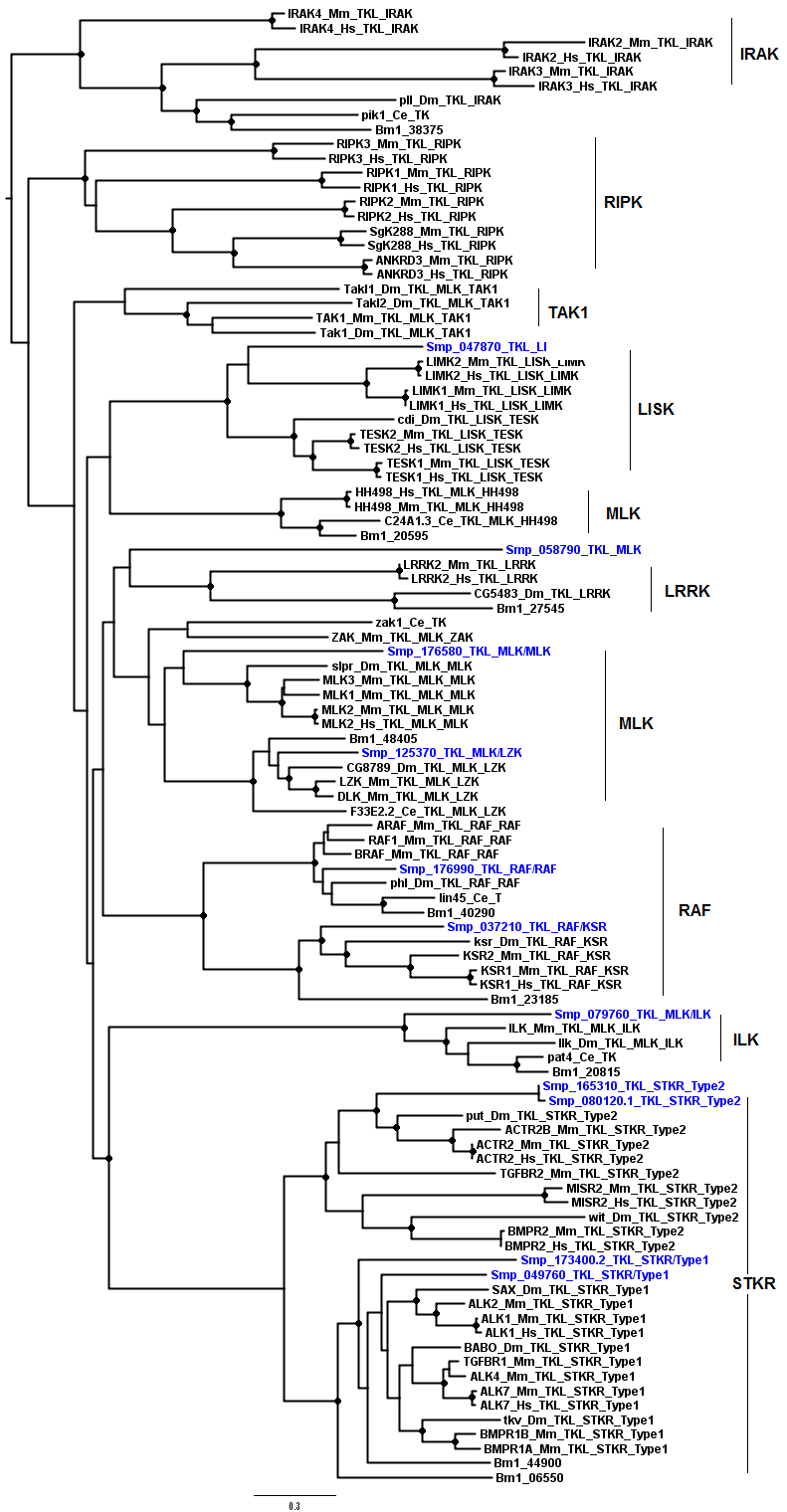

Supplement: Additional file 9 — Phylogenetic analysis of the TKL group. Amino acid sequences of the catalytic domain of the CK1 proteins of S. mansoni (blue labels), C. elegans, D. melanogaster, S. cerevisiae, H. sapiens, and B. malayi were used to construct a distance-based phylogenetic tree using PHYLIP programs. Bootstrap values (1000 replicates) equal or higher than 80% are indicated (●) as cutoff values to support the family/subfamily classification. Functional classification is indicated individually in the protein labels. Protein families are highlighted by the vertical bars and include: IRAK (IL1 Receptor Associated Kinase), RIPK (Receptor Interacting Protein Kinases), LISK (Family containing closely related TESK and LIMK sub-families), MLK (Miked Lineage Kinases), LRRK (Leucine Rich Repeat Kinase), RAF, STKR (Serine/Threonine Kinase Receptors - receptors for activin and TGFb ligands). [file 1471-2164-12-215-S9.PNG]
